# Longitudinal profiling reveals a persistent intestinal dysbiosis triggered by conventional anti-tuberculosis therapy

**DOI:** 10.1186/s40168-017-0286-2

**Published:** 2017-07-07

**Authors:** Sivaranjani Namasivayam, Mamoudou Maiga, Wuxing Yuan, Vishal Thovarai, Diego L. Costa, Lara R. Mittereder, Matthew F. Wipperman, Michael S. Glickman, Amiran Dzutsev, Giorgio Trinchieri, Alan Sher

**Affiliations:** 1 Immunobiology Section, Laboratory of Parasitic Diseases, National Institute of Allergy and Infectious Diseases, National Institutes of Health, Building 33, Room 1W10A, 33 North Drive, MSC 3202, Bethesda, MD 20892-3202 USA; 2 Cancer and Inflammation Program, Leidos Biomedical Research, Inc., Frederick National Laboratory for Cancer Research, Frederick, MD USA; 3 Immunology Program, New York, NY USA; 4 Infectious Diseases Service, Department of Medicine, Memorial Sloan Kettering Cancer Center, New York, NY USA; 5 Clinical and Translational Science Center, Weill Cornell Medical College, New York, NY USA; 6 Weill Cornell Medical College, New York, NY USA; 7 Cancer and Inflammation Program, Center for Cancer Research, National Cancer Institute, National Institutes of Health, Bethesda, MD USA; 8 Present Address: Center for Innovation in Global Health Technologies, Northwestern University, Evanston, IL USA

**Keywords:** Microbiota, Tuberculosis, Antibiotics, Dysbiosis, 16S rRNA

## Abstract

**Background:**

Effective treatment of *Mycobacterium tuberculosis* (*Mtb*) infection requires at least 6 months of daily therapy with multiple orally administered antibiotics. Although this drug regimen is administered annually to millions worldwide, the impact of such intensive antimicrobial treatment on the host microbiome has never been formally investigated. Here, we characterized the longitudinal outcome of conventional isoniazid-rifampin-pyrazinamide (HRZ) TB drug administration on the diversity and composition of the intestinal microbiota in *Mtb*-infected mice by means of 16S rRNA sequencing. We also investigated the effects of each of the individual antibiotics alone and in different combinations.

**Results:**

While inducing only a transient decrease in microbial diversity, HRZ treatment triggered a marked, immediate and reproducible alteration in community structure that persisted for the entire course of therapy and for at least 3 months following its cessation. Members of order Clostridiales were among the taxa that decreased in relative frequencies during treatment and family Porphyromonadaceae significantly increased post treatment. Experiments comparing monotherapy and different combination therapies identified rifampin as the major driver of the observed alterations induced by the HRZ cocktail but also revealed unexpected effects of isoniazid and pyrazinamide in certain drug pairings.

**Conclusions:**

This report provides the first detailed analysis of the longitudinal changes in the intestinal microbiota due to anti-tuberculosis therapy. Importantly, many of the affected taxa have been previously shown in other systems to be associated with modifications in immunologic function. Together, our findings reveal that the antibiotics used in conventional TB treatment induce a distinct and long lasting dysbiosis. In addition, they establish a murine model for studying the potential impact of this dysbiosis on host resistance and physiology.

**Electronic supplementary material:**

The online version of this article (doi:10.1186/s40168-017-0286-2) contains supplementary material, which is available to authorized users.

## Background

Tuberculosis (TB) is now the leading cause of death by a single infectious disease. In 2015, WHO estimated a third of the global population to be latently infected with *Mycobacterium tuberculosis* (*Mtb*) with 10.4 million active TB cases and 1.4 million deaths annually [1]. Effective treatment of drug-susceptible TB requires at least 6 months of daily therapy with multiple orally administered antibiotics, making it one of the longest courses of antibiotic therapy required to treat an infectious disease. Moreover, multiple-drug resistant (MDR) TB may require up to 2 years of daily therapy with more toxic and expensive second-line antibiotics. This long treatment duration sets the stage for lack of compliance, therapeutic failures, and/or relapse that can promote continued transmission as well as drug resistance.

Isoniazid (INH), rifampin (RIF), pyrazinamide (PZA), and ethambutol (EMB) are the four drugs in the first-line antimicrobial regimen used clinically to treat drug-susceptible TB [1]. While INH, PZA, and EMB are thought to specifically target mycobacteria, RIF is a broad-spectrum antibiotic with potency against many gram-positive and gram-negative bacteria [2–5]. For this reason, one might predict the standard multidrug TB treatment to have a wide range of effects on the commensal flora as has been documented with other antibiotic treatments [6–17]. Nevertheless, it is unclear if the major mycobacteria targeted antibiotics in the drug cocktail have unexpected effects on the microbiome either on their own, in combination with each other, or with rifampin itself.

The intestinal microbiota is now known to have a major influence on a range of nutritional, metabolic, and immunological processes [18–29]. The mammalian gut microbiota consists predominately of bacteria of the phyla Bacteroidetes and Firmicutes with the phyla Proteobacteria, Actinobacteria, Verrucomicrobia, and Fusobacteria together contributing a smaller fraction [30]. Alterations in the composition of the gut flora have been associated with intestinal disorders such as inflammatory bowel disease (IBD) as well as extra-intestinal and systemic conditions such as obesity, diabetes, allergies, rheumatoid arthritis, autism, and Parkinson’s disease [31–36]. As noted above, many antibiotics are known to alter the normal composition of the microbiota resulting in a state of dysbiosis, which can be either short-term or persistent. Often in the latter situation, the original bacterial diversity is largely restored following cessation of treatment with the composition of species indefinitely altered [37, 38]. These effects and their functional consequences are particularly striking in the case of neo-natal antibiotic exposure which in mice has been shown to suppress intestinal Th17 responses, promote asthma, and induce metabolic changes leading to an increased body mass index [39–41]. The latter observations in murine models are supported by parallel clinical studies in which pre-school children receiving antibiotics were shown to be more susceptible to asthma and adult obesity [42–44].

In adults, there is evidence that at least some of the side effects of antibiotics including altered metabolism and absorption of nutrients, celiac-like syndrome, colitis, and antibiotic-associated diarrhea [12] are associated with changes in the intestinal flora. In cancer studies, pre-treatment of mice with broad-spectrum antibiotics decreases the efficacy of the anti-cancer therapy, an outcome that has been linked to the role of specific commensal bacteria in the anti-tumor immune response [45–48]. A further consequence of antibiotic perturbation of the microbiota is loss of resistance to pathogens [12, 49]. For example, treatment with clindamycin, which is associated with a marked loss of diversity in the commensal flora results in increased and long-lasting susceptibility to *Clostridium difficile* colitis in both mice and humans [50]. Similarly, treatment with vancomycin and to a lesser extent metronidazole can also increase susceptibility to *C. difficile* and allows dense colonization of vancomycin-resistant *Enterococcus*, *Klebesiella pneumoniae*, and *Escherichia coli* [37, 38]. In the case of pulmonary mycobacterial infection, a recent study reported increased susceptibility to *Mtb* challenge in mice treated with broad-spectrum antibiotics [51]. Surprisingly, despite these extensive studies on antibiotics used in routine antibacterial therapy, there is only sparse information on the effects of the widely deployed conventional TB antibiotics on both the composition of the microbiota [52] and the possible physiological consequences of its alteration.

In this study, we have characterized the changes induced by front-line TB antibiotics on the composition of the intestinal microbiota in a murine *Mtb* infection model. We re-examine previous work documenting a minor influence of experimental *Mtb* infection on the murine intestinal flora and then perform extensive longitudinal analyses of the impact of anti-tuberculosis therapy (ATT) on the microbiota composition. Our findings reveal a global alteration of the commensal bacterial taxa that is detectable as early as 1 week after the start of treatment. Importantly, many of the observed compositional changes in the microbiota persist for months following cessation of therapy. Further analysis revealed that the observed changes result from the synergistic action of the different components of the antibiotic cocktail, with RIF having the most profound effect on the outcome of treatment. These findings that strikingly parallel the observations in a companion clinical study (Wipperman et al., in revision) indicate that a highly defined dysbiosis in the intestinal flora is an important sequela of conventional TB therapy.

## Results

### Murine *Mtb* infection induces only minor changes in the intestinal microbiota

To characterize the changes in the intestinal microbiota during murine *Mtb* infection and treatment, we performed an 8-month longitudinal study using C57BL/6 mice and an antibiotic administration protocol commonly employed in TB drug studies that closely mirror conventional human ATT [53]. Two groups of mice were infected with *Mtb* (H37Rv) by aerosol inhalation and a third group (“*naïve*”) served as the uninfected age-matched control. One of the infected groups of mice (“*TB + HRZ*”) was started on HRZ treatment 4 weeks’ post infection (W4) and switched to HR 2 months (W12) into therapy (Additional file 1: Figure S1). The other infected group (“*TB*”) was left untreated. Stool pellets were collected 1 week post infection and at regular intervals thereafter. After 4 months of treatment, therapy was terminated and stool samples were collected for an additional 3 months (“*post HRZ*”) (Additional file 1: Figure S1). We employed 16s rRNA (V3–V4 region) sequencing to analyze the composition of the microbiota in the stool samples.

**Fig. 1:**
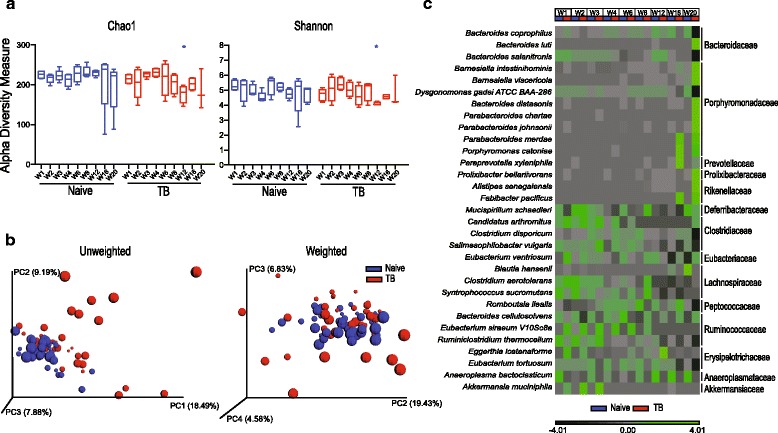
*Mtb* infection causes minimal alterations in the diversity and composition of the intestinal microbiota. **a** Alpha diversity estimates calculated from the sequenced data using Chao1 (*left panel*) and Shannon (*right panel*) indices for each time point (W1–W20) of stool sample collection in the *naïve* and *TB* group. Fecal collection time points are shown along the *x*-axis. *Error bars* indicate minimum and maximum values. Statistical significance was calculated between the corresponding time points of the two groups. *p < 0.05 (Wilcoxon rank-sum test). **b** Principal coordinate (PC) analysis of unweighted (*left*) and weighted (*right*) UniFrac distances of the microbial sequence data in the two animal groups. Each sphere represents a single animal with the size of the sphere referring to the sample collection time point (early to late time points indicated as a gradient in the size of the spheres from small to large). One sample each from W16 and W20 time points of the *naïve* group was not included in the analysis since these two samples formed an independent cluster highly separated from and inconsistent with the other clusters (For comparison, these samples are included, nevertheless, in Additional file 13: Figure S13). **c** Heat map comparing average abundances of species level classification of the 16S sequences from the *naïve* and *TB* animal groups. Data are clustered according to sample collection time point and animal group along the *x*-axis. The species indicated on the *y*-axis are grouped according to family level classification as noted on the right of the map and were filtered for those with an overall relative variance >3 (see “Methods”). *n* = 4–5 except *TB* group W20 time point where *n* = 3

To determine the changes in the intestinal microbiota due to *Mtb* infection in this particular experimental setting, we first performed a longitudinal comparison of the microbiota of the mice from the untreated *naïve* and *TB* groups. When data from all of the time points were pooled, we did not observe a statistically significant change in diversity resulting from *Mtb* infection as assessed by Chao1 and Shannon indices (Additional file 2: Figure S2a) which measure the total number of operational taxonomic units (OTUs) and, in case of the Shannon index, the richness, abundance, and evenness of the OTU distribution. However, a slight but significant decrease in diversity was evident at W12 of infection (Fig. 1a). We then used the phylogeny based UniFrac method to compare the bacterial communities in the *naïve* versus *TB* animals (Fig. 1b). Although unweighted UniFrac analyses, which cluster the data based on presence or absence of OTUs, clustered the *naïve* and *TB* samples separately (*p* < 0.001), the clustering driven by *Mtb* infection was not statistically significant based on weighted UniFrac distances (*p* = 0.203) that also take into account the relative abundance of the OTUs.

We next compared the composition of the microbiome to identify bacterial taxa that differ between the two groups. In agreement with Winglee et al. [54], we observed trends of differential abundance in members of the order Clostridiales of phylum Firmicutes and certain members of phyla Bacteroidetes and Tenericutes between the two groups (Fig. 1c, Additional file 3: Figure S3). Nevertheless, none of these differences, except genus *Alkaliphilus* that was increased in *naïve* mice, remained significant over the entire duration of the experiment. Together, these findings involving our specific infection and animal housing conditions and one inbred host genetic background, while distinct in detail from the previously published data, confirm that *Mtb* exposure by itself causes only minor changes in the composition of the murine intestinal flora.

### Anti-tuberculosis therapy induces a rapid alteration in the microbiota that persists during treatment

**Fig. 2:**
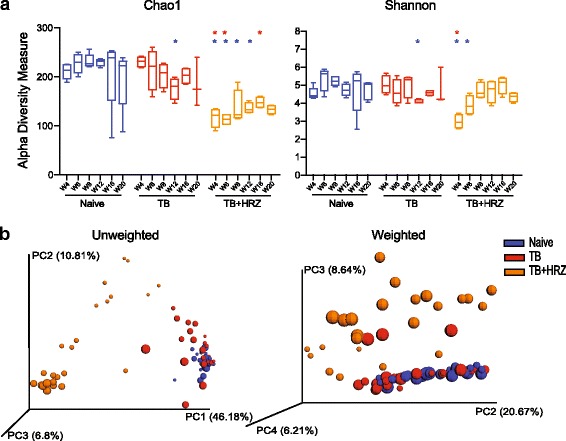
ATT changes the intestinal bacterial community structure. **a** Community diversity in the *naïve*, *TB*, and *TB + HRZ* animal groups calculated from 16S sequences (W4–W20) using Chao1 (*left*) and Shannon (*right*) indices. Fecal collection time points are shown along the *x*-axis. *Error bars* indicate minimum and maximum values. Significance tests were performed between the corresponding time points in the naive and each experimental group (*TB* or *TB + HRZ*) and in a separate comparison between the *TB* and the *TB + HRZ* groups. Significant differences with respect to *naive* or *TB* are marked with a blue or red asterisks. *p < 0.05 (Wilcoxon rank-sum test). **b** Principal coordinate (PC) analysis of unweighted (*top*) and weighted (*bottom*) UniFrac distances of the sequences from the three groups. Sizes of spheres depict the time of sample collection as described in Fig. 1b. One sample each from W16 and W20 time points of the *naïve* group was not included in the analysis since these two samples formed an independent cluster separated from and inconsistent with the other clusters (For comparison, these samples are included, nevertheless, in Additional file 13: Figure S13)

To address the primary question of this study, we examined the effects of antibiotic treatment on the microbiota composition in *Mtb* infected mice in a three-way comparison of the *naïve*, *TB* and *TB + HRZ* groups using the same methodology described above. Beginning with an analysis of data pooled from all time points of each of the groups, we found that antibiotic treatment causes a significant decrease in bacterial diversity (Additional file 2: Figure S2b). When analyzed temporally, the Chao1 index indicated statistically significant lower numbers in bacterial OTUs over multiple time points versus both the age-matched *naïve* and *TB* controls. However, the Shannon index demonstrated that the loss of microbial diversity was transient and significant only during the first 2 weeks of treatment (Fig. 2a). Taken together, these two analyses suggest that while the total number of OTUs fails to recover over the course of treatment, the evenness (i.e., inner proportion) of the OTUs does rebound by 2 weeks.

**Fig. 3:**
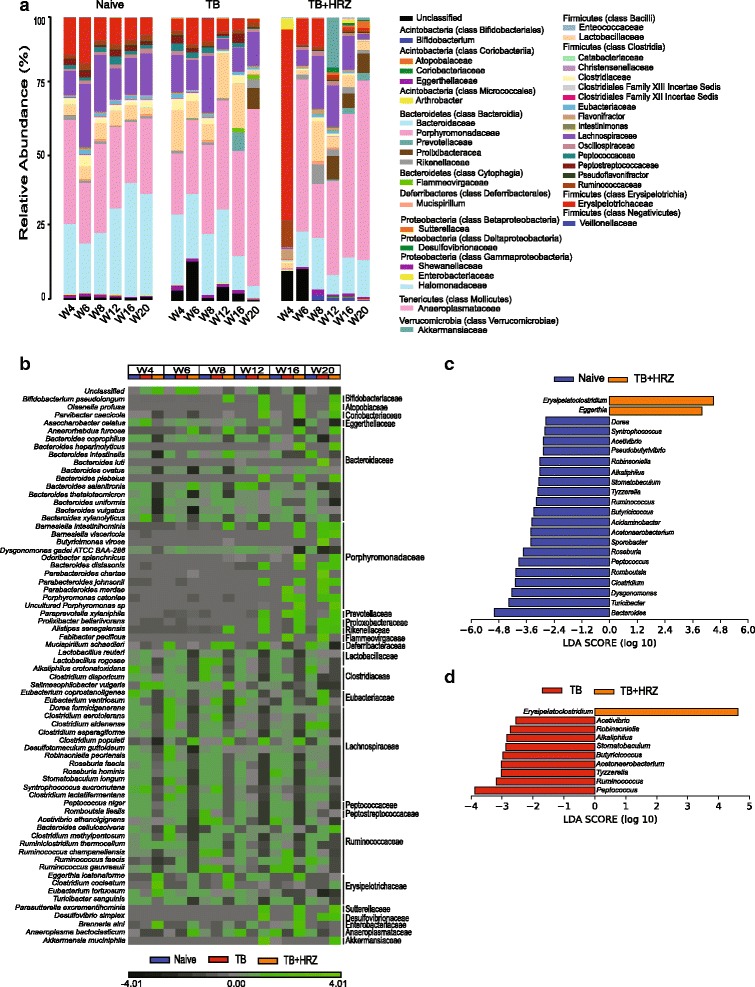
ATT causes a rapid and persistent alteration in the composition of the intestinal microbiota. **a** Average relative abundance of bacterial families in each group and experimental time point identified from the sequenced data of the stool samples. Time points are indicated along the *x*-axis and arranged by the experimental groups. The bacterial families are grouped under their respective phylum and class in the color key. **b** Heat map showing the average species level abundance. Data are filtered for overall relative variance >5 and clustered as described in Fig. 1c. **c**, **d** LEfSe analyses depicting genera that are differentially abundant between the *naïve* and *TB + HRZ* groups (**c**) and *TB* and *TB + HRZ* groups (**d**). Analyses were performed on data from W4 to W20 time points (see “Methods”). Taxa significantly enriched in *naïve*, *TB*, and *TB + HRZ* groups are shown with *blue*, *red*, and *orange bars*, respectively. Data are filtered for *p* < 0.01 and linear discriminant analysis (LDA) score >2. *n* = 4–5 except *TB* group W20 time point where *n* = 3

We next compared the overall community structure and composition of the bacteria in the three groups. Unweighted and weighted UniFrac analyses revealed a highly separated clustering of samples collected from *TB + HRZ* mice from those of both the *naïve* and *TB* animals (unweighted, *p* < 0.001 for both comparisons; weighted, *naïve* versus *TB + HRZ p* < 0.001; *TB* versus *TB + HRZ p* < 0.01) (Fig. 2b). Indeed, in this three-way analysis, the samples from *naïve* and *TB* mice clustered together, re-enforcing the finding that infection does not cause a major alteration of the intestinal flora. HRZ treatment, however, separated treated and untreated samples with a variance of 46.18% being described on the first coordinate in the unweighted UniFrac analysis and a variance of 8.64% on the third coordinate in weighted UniFrac analysis. As might be predicted, the bacterial composition of the stool samples from the *TB + HRZ* mice showed highly significant differences in comparison to that of the mice from *naïve* and *TB* groups (Fig. 3, Additional file 3: Figure S3b).

When data from all time points were grouped and compared, HRZ treatment resulted in significantly decreased relative frequencies of genera *Acetivibrio*, *Robinsoniella*, *Alkaliphilus*, *Stomatobaculum*, *Butyricicoccus*, *Acetanaerobacterium*, *Tyzzerella*, *Ruminococcus*, and *Peptococcus* all belonging to the class Clostridia of the phylum Firmicutes (Fig. 3c–d). Additional decreases in genera, mostly belonging to class Clostridia, were evident in the specific comparison of *naïve* mice to *TB + HRZ* animals (Fig. 3c). Members of Actinobacteria, the phylum under which *Mycobacterium* is classified, did not show any significant alterations overall as a result of treatment. Interestingly, increases (as opposed to decreases) in bacterial taxa due to HRZ treatment were limited to genus *Erysipelatoclostridium* (and genus *Eggerthia* in the *naïve* versus *TB + HRZ* comparison).

**Fig. 4:**
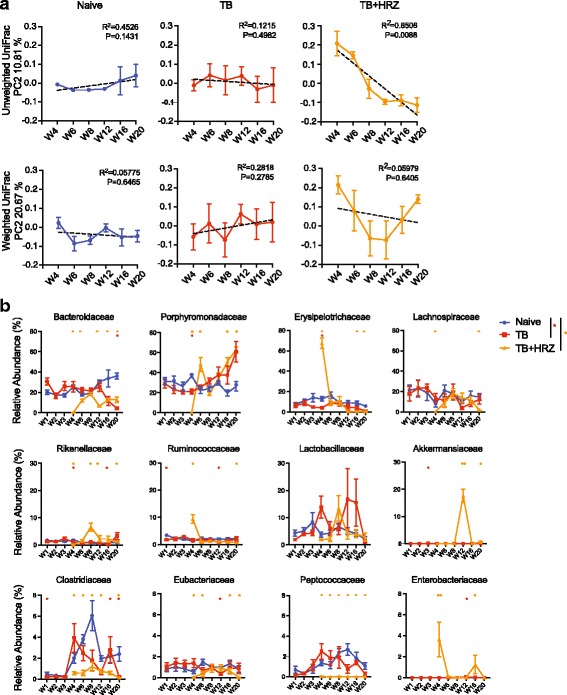
ATT induces temporal changes in the community structure of the intestinal flora. **a** Principal coordinate (PC) values along the second ordinate of unweighted (*top*) and weighted (*bottom*) UniFrac analyses shown in Fig. 2b for each sample of the three animals groups are plotted. The *x*-axes indicate the sample collection time points and the *y*-axes indicate the PC values. The *dotted lines* represent the linear regression analysis trends. *Error bars* indicate mean ± SEM. **b** Relative abundances from W4 to W20 of select bacterial families for the three animal groups are plotted. *p < 0.05; **p < 0.01 (Wilcoxon rank-sum test). *Error bars* indicate mean ± SEM. *n* = 4–5 except *TB* group W20 time point where *n* = 3

Having demonstrated that HRZ induces highly significant alterations in the bacterial composition of the intestinal microbiome, we went on to describe these changes longitudinally. Both unweighted and weighted UniFrac analyses revealed a longitudinal separation of the samples along the second coordinate (Fig. 2b) suggesting a temporal change in community structure. Furthermore, linear regression analyses revealed a statistically significant trend in the change in community structure over time in the *TB + HRZ* but not in the *naïve* or *TB* groups (Fig. 4a). Interestingly, analysis of the *TB + HRZ* group data using the principle coordinates from the weighted UniFrac analysis showed a significant trend only up to W12 (Fig. 4a, trend line not shown, *R* ^2^ = 0.9095, *p* = 0.0463 based on regression analysis up to W12). The subsequent change occurred at the first time point of stool collection (W16) following the switch in the antibiotic regimen from triple HRZ to double HR administration.

Longitudinal analysis revealed a dramatic alteration in the bacterial composition occurring 1 day (indicated as W4 in Additional file 1: Figure S1) following start of HRZ treatment (Figs. 3a–b, 4b). This change was evident in both the alpha diversity (Fig. 2a) and UniFrac analyses (Fig. 2b, smallest spheres). By 2 weeks post treatment (W6), changes in bacterial composition due to antibiotic administration stabilized and persisted with minor fluctuations for the remainder of the treatment period. These consisted of a transient increase in genus *Akkermansia* at W12 and W20 and increases in genera *Barnesiella*, *Paraprevotella*, *Bifidobacterium*, and *Porphyromonas* of phylum Bacteroidetes and certain members of phylum Actinobacteria at later time points of treatment (Fig. 4b, Additional file 4: Figure S4, Additional file 5: Figure S5). In addition, family Erysipelotrichaceae showed a dramatic increase on day 1 (W4) after start of therapy, and while decreasing over time in the treated mice remained significantly high in comparison to age-matched *naïve* and *TB* mice and interestingly, certain members of this family showed an increase while others decreased (Fig. 3b). Transient increases were also observed in members of the order Enterobacteriaceae and other members of the phylum Proteobacteria (Fig 4b, Additional file 4: Figure S4, Additional file 5: Figure S5).

The changes in bacterial community structure and composition induced by antibiotic treatment were comparable in both *Mtb* infected and uninfected animals and were reproducible in two independent experiments comparing *Mtb* infected mice that were either treated or untreated (Additional file 6: Figure S6, Additional file 7: Figure S7). Together, these observations revealed that treatment with conventional anti-tuberculosis drugs causes a transient decrease in the diversity of the intestinal microbiota along with persistent, fluctuating changes in its composition and that this dysbiosis does not appear to be influenced by the presence or absence of *Mtb* infection.

### The dysbiosis induced by ATT is maintained after cessation of treatment

**Fig. 5:**
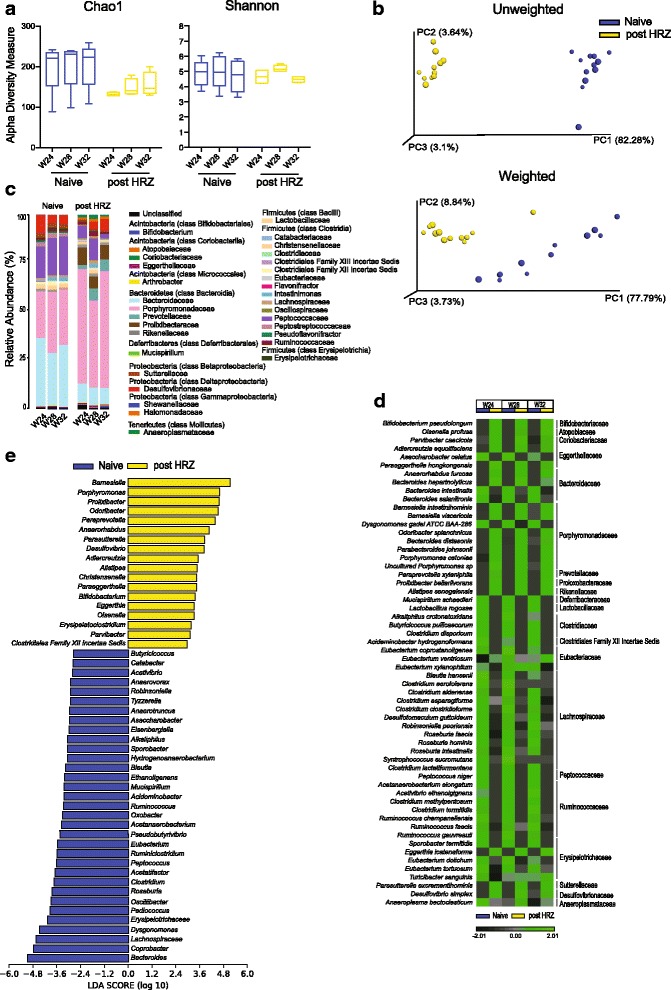
The intestinal dysbiosis triggered by ATT is long-lasting and persists following cessation of treatment. **a** Community diversity in the *naïve* and *post HRZ* animal groups calculated from 16S sequences (W24–W32) using Chao1 (*left*) and Shannon (*right*) indices. *Error bars* indicate maximum and minimum values. **b** Principal coordinate (PC) analysis of unweighted (*top*) and weighted (*bottom*) UniFrac distances of the sequences from the two groups. Sizes of spheres depict the time of sample collection as described in Fig 1b. One sample each from W24, W28, and W32 time points of the *naïve* group was not included in the analysis since these three samples formed a separate independent cluster inconsistent with the other clusters. (For comparison, these samples are included, nevertheless, in Additional file 13: Figure S13). **c** Average relative abundance of bacterial families identified from the sequenced data of the *naïve* and *post HRZ* stool samples (W24–W32). The bacterial families are grouped under their respective phylum and class in the color key. **d** Heat map showing the average species level relative abundance. Data are filtered for overall relative variance >5 and clustered as described in Fig. 1c. **e** LEfSe comparisons showing the differentially abundant genera between the *naïve* and *post HRZ* groups (W24–W32). Taxa significantly enriched in *naïve* or *post HRZ* groups are depicted with *blue* or *yellow* bars, respectively. Data are filtered for *p* < 0.05 and LDA score >2. *n* = 4–5

To determine whether a standard regimen of anti-tuberculosis therapy has long-term effects on the microbiota, we monitored bacterial populations in stool samples at monthly intervals for 3 months’ post cessation of therapy (*post HRZ*) in comparison with samples from age-matched *naïve* animals (Additional file 1: Figure S1). No significant increase in the diversity of the microbiota was observed during this period following removal of antibiotic pressure (Fig. 5a, Additional file 2: Figure S2c). Importantly, UniFrac analysis revealed that the *post HRZ* microbiota continued to cluster separately from the bacterial populations present in the *naïve* samples (Fig. 5b) closely overlapping with the cluster formed from the data of the actively treated *TB + HRZ* group at latter time points (Additional file 8: Figure S8a).

A more detailed compositional analysis confirmed the close similarity of the microbiota in mice from the *TB + HRZ* and *post HRZ* groups (Additional file 8: Figure S8b) with a few notable exceptions. Members of the family Erysipelotrichaceae that had increased in the presence of antibiotics decreased following cessation of therapy but remained at a frequency significantly higher than that observed in age-matched *naive* controls (Fig. 5c–e). Genus *Lactobacillus* that did not show a significant decrease during active treatment decreased following cessation of therapy (Additional file 8: Figure S8b) while relative levels of genera *Barnesiella*, *Porphyromonas*, and *Paraprevotella* of phylum Bacteroidetes and genera *Parasutterella* and *Desulfovibrio* of phylum Proteobacteria were further increased during the same period (Fig. 5e). Interestingly, the relative frequencies of many members of Actinobacteria were increased post treatment (Fig. 5d, e). At the phylum level, the Bacteroidetes/Firmicutes ratio was 60.2/37.8 (%) in the *naive* group versus 77.2/17.3 (%) in the *post HRZ* group. Additionally, annotation of metagenome function based on the 16S sequence data using the Greengenes database [55] and PICRUSt metagenome prediction tool [56] suggests a difference in the coding capacity of the microbiome in the post treatment mice in comparison to age-matched controls (Additional file 9: Figure S9). Of note, the coding capacity associated with carbohydrate metabolism is decreased and with energy metabolism increased in microbiota from HRZ-treated mice. Such differences in metabolic activity, particularly in terms of short-chain fatty acid levels, have previously been implicated in a number of homeostatic host functions [57]. Together, these findings indicated that ATT triggers a dysbiosis that maintains its basic compositional structure long after cessation of antibiotic treatment despite alterations in certain taxa.

### The dysbiosis induced by multi-antibiotic therapy results primarily from the synergistic effects of rifampin and pyrazinamide

**Fig. 6:**
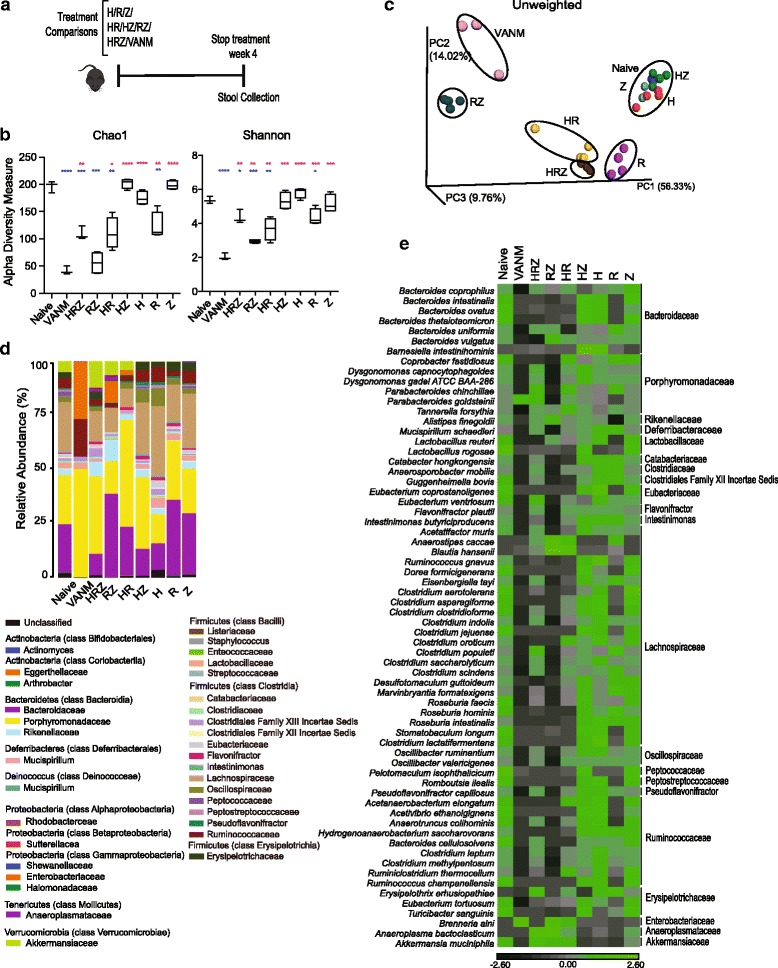
Differential effects of the components in the HRZ cocktail on the intestinal microbiota. **a** Nine groups of mice with 3–4 animals in each group were employed. One group was left untreated as the naïve age-matched control. Seven of the groups were each treated with one or a combination of H (Isoniazid), R (Rifampin), and/or Z (Pyrazinamide) as indicated and separated by a ‘/’. The last group was treated with a cocktail of vancomycin, ampicillin, neomycin, and metronidazole (VANM). **b** Bacterial community diversity of all the samples in each group was estimated using alpha diversity indices Chao1 (*left*) and Shannon (*right*). *Error bars* indicate maximum and minimum values. *p < 0.05; **p < 0.01; ***p < 0.001; ****p < 0.0001, Welch’s *t* test. Blue and pink asterisks indicate significance in comparison to Naïve and VANM, respectively. **c** Principal coordinate (PC) analysis of unweighted UniFrac distances of sequences from all nine groups. **d** Average relative abundance of bacterial families in each group identified from the sequenced data. The bacterial families are grouped under their respective phylum and class in the color key. **e** Heat map showing the average species level abundance. Data shown are filtered for an overall relative variance >10 and depicted as described in Fig. 1c except along the *x*-axis which show the different treatment groups. Naïve, VANM, HRZ, *n* = 3; remaining groups *n* = 4

Having described the major effects of the antibiotic cocktail used in ATT on the intestinal microbiota, we next sought to determine which drugs in the cocktail were responsible for the changes observed. To do so, we treated uninfected and 4-week *Mtb*-infected mice with each of the three antibiotics individually and in combinations of two and compared the outcome with that occurring in mice receiving the complete triple cocktail (Fig. 6, Additional file 10: Figure S10). To assess the extent of the alterations induced, we included an additional group consisting of uninfected mice receiving vancomycin, ampicillin, neomycin, and metronidazole (VANM), the combination treatment used routinely for depletion of the commensal intestinal flora [29, 58]. Stool samples from these animal groups were collected at a single time point (4 week) into drug treatment (Fig. 6a).

Consistent with its broad-spectrum antibiotic activity, RIF but not INH or PZA caused a decrease in bacterial diversity in uninfected mice which was comparable to that triggered by the complete HRZ cocktail (Fig. 6b). Similarly, RIF alone induced major compositional changes in the microbiota but these alterations were distinct in terms of the specific taxa affected and/or their magnitude from those observed following HRZ treatment (Fig. 6c–e, Additional file 11: Figure S11). For example, RIF-treated mice showed increases in certain Clostridia species that did not occur in HRZ-treated animals (Fig. 6e). Unexpectedly, despite their well-known targeted activity against mycobacteria and failure to induce changes in microbiota diversity (Fig. 6b), treatment with INH or PZA alone nevertheless triggered quantitative compositional alterations (Additional file 11: Figure S11). For example, INH caused increases in *Barnesiella* and PZA in genus *Anaeroplasma* of phylum Tenericutes and both drugs affected the relative levels of certain Clostridia species. Interestingly, some of these alterations did not mirror the trends observed with the complete HRZ cocktail.

Examining the effects of dual antibiotic administration, drug pairs in which RIF was one of the partners as expected induced the only significant decreases in diversity (Fig. 6b). Nevertheless, all three pairs tested caused major compositional changes and these were again at the community level distinct from those induced by the entire HRZ cocktail (Fig. 6c–e). For example, HRZ treatment did not significantly affect levels of *Brenneria* (Enterobacteria), whereas this genus showed a significant increase following dual RZ (and to a lesser extent HR) treatment (Additional file 11: Figure S11), which was comparable to that observed following VANM administration. Neither RIF nor PZA alone caused a significant increase in Enterobacteria.

Unexpectedly, in terms of relative frequencies, HRZ induced many of the same compositional changes observed following VANM antibiotic treatment (Fig. 6e, Additional file 12: Figure S12). However, striking differences were observed in the Proteobacteria, which underwent a greater relative increase following VANM versus HRZ administration, and in *Erysipelatoclostridium*, which increased following HRZ but not VANM treatment. Interestingly, except for differences in some members of the order Bacteroidales and Clostridiales, the compositional changes due to RZ dual treatment (as opposed to HRZ) closely resembled those caused by VANM (Fig. 6e, Additional file 12: Figure S12) as evident from the tighter clustering of the data from these mice in UniFrac analysis (Fig. 6c).

In summary, the above experiments indicated that each of the antibiotic components of the HRZ cocktail contributes to the overall dysbiosis occurring following drug treatment of uninfected mice, with RIF and PZA having the most prominent effects. The results of parallel experiments using infected as opposed to uninfected mice confirmed this main conclusion with only minor differences evident in the individual taxa affected (Additional file 10: Figure S10). These minor changes may relate to the absence of the *Mtb*-associated enzymes required for drug activation in the uninfected animals.

## Discussion

Despite the growing number of studies describing the impact of antibiotic treatment on the intestinal microbiota, to the best of our knowledge, the effects of the drugs used in conventional anti-tuberculosis therapy including the broad-spectrum antibiotic rifampin have not been previously characterized. Our longitudinal studies on the stool microbiota demonstrate that treatment of *Mtb-*infected mice with HRZ using a regimen similar to that employed in patients results in a rapid but transient loss in bacterial diversity as well as persistent alterations in the microbiome composition that do not recover following cessation of therapy. Our findings also reveal that these changes are largely independent of the mycobacterial infection itself and that they depend on the combined effects of the different components in the triple antibiotic cocktail.

In agreement with previous studies using a different mouse strain and 16S rRNA sequencing strategy [54], we found that infection with *Mtb* H37Rv causes distinct alterations (largely focused on the members of the order Clostridiales) in the composition of the gut microbiota. However, these changes were minor in magnitude and scope when compared to those observed following TB antibiotic treatment. We based the latter conclusion largely on the UniFrac analysis, which revealed that sequences from uninfected and *Mtb*-infected animals cluster together and separately from those obtained from antibiotic-treated mice. In agreement, we found that in the comparison of uninfected-untreated (*naïve*) to infected-treated (*TB + HRZ*) animals and in the comparison of infected-untreated (*TB*) to infected-treated (*TB + HRZ*) animals, the majority of the taxa altered as a consequence of treatment are identical. Further, a number of the taxa identified in the above analysis were also altered in a separate comparison to treated but uninfected animals (Additional file 11: Figure S11).

The absence of major *Mtb* induced changes in the gut microbiota is not entirely surprising given that the intestinal tract is not the primary site of infection for this pathogen. In contrast, one might expect an effect of orally administered antibiotics on the microbiota. Indeed, many of the taxa altered during HRZ treatment were also affected by the VANM antibiotic cocktail that is used routinely as a tool for depleting the intestinal microbiome. Nevertheless, we observed some striking differences in the effects of HRZ and VANM in our treated mice (e.g., on Erysipelotrichaceae and Enterobacteriaceae). Moreover, VANM triggers a major decrease in microbial diversity not seen in HRZ-treated mice. Although not yet directly investigated by us, these observations suggest that if HRZ treatment has any immunological consequences, they will be distinct from those triggered by VANM.

An important part of the present study was to compare the effects on the microbiota of the individual drugs administered together in the HRZ cocktail. Surprisingly, both INH and PZA, which are prodrugs [4, 59], triggered statistically significant decreases in the relative abundance of species with no obvious phylogenetic relationship to the mycobacteria which they are meant to target. We also observed that the RZ cocktail induced alterations in the microbiota that exceeded those triggered by HRZ. This increased potency of RZ versus HRZ on the microbiota is consistent with the known efficacy of these drug combinations against *Mtb* itself which is thought to be related to the negative effects of INH on RIF availability [60]. A larger inference of these findings is that the presence of INH may be a dampening force that limits the potential severity of the dysbiosis induced by the entire TB drug treatment cocktail.

The fascinating “crosstalk” between antibiotics in the targeting of the microbiota that we document here has been observed in other settings [38] and is not well understood. In addition to possible interactions in the mechanism of action of the drugs themselves, individual antibiotics could indirectly affect the survival of unrelated bacterial taxa by altering their biological niches. Such indirect influences may also explain the unexpected effects of INH and PZA on the non-mycobacterial species, we observed following treatment with these antibiotics. Whether or not this outcome reflects the presence in the gut microbiota of cryptic mycobacterial species or unrelated bacteria sharing the same drug target and/or activating enzyme remains to be determined. Although not employed in the present study or in many publications involving murine TB drug treatment models [53, 61], the mycobacteria-targeted drug ethambutol is a mainstay of the antibiotic cocktail used to treat clinical TB. In the light of our findings that mycobacteria-specific antibiotics can also alter the composition of the gut microbiome, it will be important to include an analysis of the effects of ethambutol in future studies in the murine model.

An important finding of our studies in the murine model is that the altered composition of the microbiota following TB antibiotic treatment persists for at least 3 months’ post cessation of therapy, a relatively long period in the life span of laboratory mice. That a similar dysbiosis occurs in TB patients receiving the same antibiotics (as well as ethambutol) is strongly supported by a companion clinical study analyzing fecal microbiota from Haitian TB patients during and post treatment (Wipperman et al., in revision). In that cross-sectional analysis, the observed dysbiosis which was found to involve many of the same taxa altered in our murine model persisted for at least a year and half and as long as 3 years following cessation of therapy, supporting the long-term nature of the dysbiotic state triggered by TB treatment.

While both our experimental model and clinical studies document the establishment of a dysbiosis induced by TB antibiotics, the impact of this state on host physiology is currently unclear. In previously published work, alterations in the microbiota have been shown to affect both host resistance to disease and immunologic function. Changes in several of the physiologically important taxa identified in previous studies were observed to be altered in our antibiotic-treated mice. For example, we documented major decreases both during and after treatment in many members of the order Clostridiales that have been previously reported to associate with altered *T*_reg_ function [62]. Similarly, we observed significant increases in Erysipelotrichaceae, changes in which have been linked with inflammatory and metabolic alterations [63–66]. On the other hand, while we did observe a statistically significant increase during treatment in Proteobacteria, a phylum containing numerous pathogenic species, the outgrowth was considerably lower than that documented following VANM treatment. The latter finding is consistent with the paucity of reports describing enteric pathogen colonization in drug-treated TB patients. In this regard, we observed a major post treatment increase in *Barnesiella*, a genus which when administered to mice was found to promote clearance of vancomycin-resistant *Enterococcus* [67]. We are currently using the murine model developed and characterized here to address whether the changes in microbiota induced by TB antibiotic treatment are associated with altered local and systemic immune responses as well as susceptibility to experimental infectious and inflammatory diseases. We are also exploring the possibility that the observed TB drug-induced shifts in the composition of the gut microbiota result in altered host metabolism thereby promoting the development of metabolic syndromes such as diabetes [68] and/or self-induced changes in the absorption of the antibiotics themselves.

The analysis presented here focused on the effects of TB antibiotics on the intestinal commensal flora. However, these drugs are orally absorbed and used to target *Mtb* in the lungs as well as other extra-pulmonary sites. Although previously thought to be largely sterile, the lung is now known to possess its own unique microbiome [69]. Thus, an important question concerns the possible effects of TB treatment on that bacterial community and its consequences for the host physiological parameters discussed above. That TB antibiotic therapy may affect the lung microbiota communities is suggested by a previous cross-sectional study in which differences in the sputum microbiota of treated versus untreated TB patients were observed [70]. Importantly, based on the findings reported here and in a companion clinical study by our colleagues (Wipperman et al., in revision), the alterations in the microbiota induced by TB drugs are likely to be long-lasting. Given the large numbers of patients who have received antibiotic treatment for tuberculosis during their lifetime, this dysbiosis may cumulatively affect millions of individuals worldwide.

## Conclusions

This study is the first to systemically address the impact of conventional anti-tuberculosis therapy on the gut microbiota. By utilizing a murine model, we have been able to control for longitudinal, age, gender, dietary, drug compliance, and other parameters not readily addressable in a clinical study. Our findings reveal that TB antibiotics cause a rapid, long-lasting dysbiosis that persists for months’ post cessation of treatment. This dysbiosis manifests as a temporal reduction in microbial diversity accompanied by profound, persistent alterations in bacterial composition, and community structure which closely resemble changes in the microbiota seen in drug-treated TB infected humans observed in a companion study. Our experiments also delineate the impact of the individual antibiotics used in TB multi-drug therapy and document unexpected interactions between them in their effects on the microbiota. In addition to revealing a major dysbiosis induced by anti-tuberculosis therapy, these findings in a murine experimental model lay important groundwork for future functional analyses of its physiological consequences.

## Methods

### Animals and housing conditions

Except where noted, all experiments were performed on 4–8-week-old C57BL/6J-CD45a(Ly5a) female mice obtained from the National Institute of Allergy and Infectious Diseases (NIAID) Taconic Farms supply contract (Germantown, NY, USA). Animals were housed at the biosafety-level 3 facility at NIAID, National Institutes of Health (NIH) and maintained on autoclaved chow and water. All experimental procedures were in compliance with protocols approved by the NIAID Animal Care and Use Committee.

### Experimental *M. tuberculosis* infection

Mice were infected with approximately 100 CFU of *M. tuberculosis* H37Rv strain via the aerosol route using a Glas Col chamber (Terre Haute, IN, USA). Successful infection was confirmed by monitoring pulmonary bacterial loads at monthly intervals by culturing tissue homogenates in 7H11 media plates supplemented with oleic acid-albumin-dextrose-catalase. Bacillary loads at 4-week post infection typically ranged between 10^5^ and 10^6^ CFU/mouse (data not shown).

### Antibiotic treatment

For TB antibiotic treatment, each mouse received 200 ul of a combination of isoniazid (25 mg/ml), rifampin (1 mg/ml), and pyrazinamide (150 mg/ml) by oral gavage 5 days a week for the first 2 months and isoniazid and rifampin only for an additional 2 months. All drugs were dissolved in water or in the case of rifampin in DMSO and then combined into stocks that were prepared freshly every week. In some experiments, the drugs were administered individually or in pairs at the same dosing indicated above. In the same experiment, one group of mice was orally gavaged 5 days a week with 200 ul of a cocktail of vancomycin, ampicillin, neomycin, and metronidazole (VANM) each dissolved in water at a concentration of 2 mg/ml except vancomycin, which was administered at a concentration of 1 mg/ml. All antibiotics were purchased from Sigma-Aldrich (St. Louis, MO, USA).

### Stool sample collection and DNA extraction

A fresh stool pellet (0.02–0.03 g) was obtained from each mouse at the time points indicated and collected into tubes pre-loaded with 2.7 mm, 1 mm, and 0.1 mm glass/zirconia beads (or in some experiments 2.7 mm metal beads) and temporarily stored at 4 ° C for 6 h or less before moving to −80 °C. At the end of each time course experiment, samples were homogenized using a Precellys homogenizer at 10,000 rpm for 1 min and total DNA extracted using the QIAamp Fast DNA Stool Mini kit according to manufacturer’s pathogen detection protocol with the following specific modification to increase DNA yield: the supernatant after heating was divided in to three equal volumes and each was processed as an independent sample until combined in the QIAmp mini spin column. The resulting purified DNA samples were stored at –20 °C prior to sequencing.

### 16s rRNA amplification and sequencing

The V3 and V4 regions of the 16 rRNA were amplified and sequenced using the Illumina MiSeq platform with the primers 5′-TCGTCGGCAGCGTCAGATGTGTATAAGAGACACCTACGGGNGGCWGCAG-3′ and 5′-GTCTCGTGGGCTCGGAGATGTGTATAAGAGACAGGACTACHVGGGTATCTAATCC-3′ as previously described [71] with the following specific conditions. The cycling parameters for the PCR amplification were 98 °C for 30 s followed by 10 cycles of touch down PCR (98 °C for 10 s, 60 °C for 30 s with a decrease by 1 °C for every cycle and 72 °C for 30 s) which was followed by 7 cycles of 98, 50, and 72 °C for 30 s each and finally 1 cycle of 72 °C for 7 min. The amplicons were then indexed using 8 PCR cycles and quantified using a KAPA library quantification kit. One hundred nanograms of DNA from each sample was used for PCR amplification, and equimolar amounts (4 nM) of each sample were pooled for sequencing. For the experiment described in Additional file 1: Figure S1, after quality control, we were able to obtain a total of 13,231,205 reads with an average of 99,482 reads per sample. For the single drug and combination drug studies, we obtained an average of 54,960 reads per sample post quality filter.

### Sequences analyses

The sequence data were processed and analyzed using the programs USEARCH (version 8.1.1831) [72] and QIIME (version 1.9.1) [73]. Chimeric reads were filtered out from the sequenced data using the Gold ChimeraSlayer reference database (version microbiomutil-r20110519) [74], and the filtered sequences that shared at least 97% pairwise nucleotide identity were binned into operational taxonomic units (OTUs). To obtain species level classification for the clustered OTUs, a custom reference database built from the NCBI 16S sequence and taxonomy database (version May 2016) was used. Taxonomic assignments were performed using the “blast” method of QIIME’s *assign_taxonomy.py* script. Alpha-diversity estimates were calculated using Chao1 and Shannon indices. Phylogeny-based unweighted and weighted UniFrac distance matrices [75, 76] were calculated using QIIME and visualized using Emperor 0.9.4 [77]. PICRUSt metagenome prediction tool [56] was used to annotate function from 16S sequence data using the Greengenes database [55]. This sequence processing and analysis work utilized the computational resources of the NIH HPC Biowulf cluster (http://hpc.nih.gov). Heat maps were generated with the Partek Genomics Suite 6.6 software (Partek Ink. St. Louis, MO, USA) using average species level OTU counts that were normalized, log-transformed, and offset by one. The data were filtered for species that were present in at least 90% of all samples with an overall relative variance as indicated in the figure legends to allow better visualization.

### Statistics

Significance tests for alpha diversity estimates were performed in QIIME using the non-parametric *t* test. Statistically significant difference between two experimental groups based on sequenced and clustered 16S data was assessed using pairwise UniFrac distances and the Adonis test with 999 permutations. The linear discriminant analysis (LEfSe) [78] was used to identify differentially abundant taxa between groups. Both Kruskal-Wallis and Wilcoxon rank-sum tests in LEfSe were used to process sample data that were first classified based on experimental group and then sub-grouped based on time to identify taxa that were altered overall irrespective of the experimental time course. Any taxa with a linear discriminant analysis (LDA) effect size >2 and *p* value <0.01 (or <0.05) was considered statistically significant. In comparisons that did not involve subgroup classification, only Kruskal-Wallis test was performed and filtered for LDA >2 and *p* value <0.05 to identify statistical significance. Significance values and any modifications in statistical tests are indicated in the text, associated figures and figure legends.

## Additional files

Additional file 1: Figure S1. Outline of experimental plan for longitudinal analysis of alterations in the microbiota induced by *Mtb* infection and/or ATT. Three groups of mice were employed each consisting of four to five animals (except the last time point of the *TB* group which consisted of three mice). For the purpose of consistency, the time points shown refer to the weeks (W) of stool sample collection relative to the date of infection rather than treatment. Fecal sample collection time points for the *naïve*, *TB*, and *TB + HRZ* groups are indicated with blue, red, and orange circles, respectively, along the experimental timeline. In the case of the *TB + HRZ* group, treatment was ceased at W20 and post treatment sampling resumed at W24 (*post HRZ* group, yellow circles). In addition to this experiment, two similarly designed experiments were performed to confirm the reproducibility of the key findings (see text and Additional file 6: Figure S6, Additional file 7: Figure S7). H, Isoniazid; R, Rifampin; Z, Pyrazinamide (PDF 352 kb)

Additional file 2: Figure S2. Analysis of bacterial community diversity in the experimental groups shown in **Figure S1.** a–c Alpha diversity estimates as calculated by Chao1 (left panel) and Shannon (right panel) indices from the 16S sequence data for each of the time points in the *naïve* and *TB* (W1–W20) (a) *naïve*, *TB*, and *TB + HRZ* (W4–W20) (b) and *naïve* (W24–W32) and *post HRZ* (W24–W32) (c). The experimental groups are indicated along the *x*-axes. The bars indicate the mean ± SEM for each animal group in the comparison. Statistical significance between the groups based on pooled data from all time points of each group was calculated using a non-parametric *t* test with 999 Monte-Carlo permutations. (PDF 408 kb)

Additional file 3: Figure S3. Relative abundance of bacterial taxa of experimental groups. a Average relative abundance of bacterial families in the W1 to W3 time points of the *naïve* and *TB* groups. Refer Fig. 3a for the remaining time points. b, c Average relative abundance of bacterial families in *naïve*, *TB*, *TB + HRZ* and *post HRZ* groups. Averages were calculated from the sequenced data of W4–W20 time points in (b) and W24–W32 time points in (c). The bacterial families are grouped under their respective phylum and class in the color key. (PDF 451 kb)

Additional file 4: Figure S4. Bacterial genera that are differentially abundant between the *naive* and *TB + HRZ* groups over time. LEfSe analysis showing the genera significantly enriched in the comparison of *naïve* versus *TB + HRZ* for the W6 to W20 stool collection time points as indicated. Genera significantly enriched in *naïve* or *TB + HRZ* groups are depicted with blue or orange bars, respectively. Data are filtered for *p* < 0.05 and LDA score >2. LEfSe analysis was performed without the “subclass” option. (PDF 656 kb)

Additional file 5: Figure S5. Bacterial genera that are differentially abundant between the *TB* and *TB + HRZ* groups over time. LEfSe analysis showing the genera significantly enriched in the comparison of *TB* versus *TB + HRZ* for the W6 to W20 stool collection time points as indicated. Genera significantly enriched in *TB* or *TB + HRZ* groups are depicted with red or orange bars, respectively. Data are filtered for *p* < 0.05 and LDA score >2. LEfSe analysis was performed without the “subclass” option. (PDF 636 kb)

Additional file 6: Figure S6. Repeat experiment demonstrating reproducibility of major differences observed during as well as post treatment. a Outline of experimental plan for longitudinal analysis of alterations in the microbiota induced by ATT in *Mtb*-infected C57BL/6J-CD45a(Ly5a) female mice. Two groups of mice (TB and TB + HRZ) were employed with each group consisting of four animals. Stool sample collection time points are indicated as colored circles (TB, red; TB + HRZ, orange). For the purpose of consistency, the time points shown refer to the month (M) of stool sample collection relative to the date of infection rather than treatment. In the case of the TB + HRZ group, treatment was ceased at M5 and post HRZ samples (yellow circles) were collected at M8. H, Isoniazid; R, Rifampin; Z, Pyrazinamide. b Community diversity in the TB and TB + HRZ animal groups for every stool sample collected was calculated from 16S sequences using Chao1 (left) and Shannon (right) indices. Error bars indicate maximum and minimum values. Significance tests were performed between the corresponding time points in the two groups. *p < 0.05, Wilcoxon-rank sum test. **c** Principal coordinate (PC) analysis of unweighted (left) and weighted (right) UniFrac distances of the sequences from the animal groups. Each sphere represents a single animal with the size of the sphere referring to the sample collection time point (early to late time points indicated as a gradient in the size of the spheres from small to large). d LEfSe analysis was performed to identify genera that are differentially abundant between the TB and TB + HRZ groups. Taxa significantly enriched in the TB or TB + HRZ groups depicted with red or orange bars, respectively. Data are filtered for *p* < 0.01 and LDA score >2. *n* = 4. (PDF 719 kb)

Additional file 7: Figure S7. Repeat experiment demonstrating reproducibility of key findings observed during treatment. See Additional file 6: Figure S6 for description with the following exceptions: C57BL/6J female mice were used and treatment was terminated at month 7 of *Mtb* infection, and mice were not monitored post cessation of therapy. *n* = 3–5. (PDF 657 kb)

Additional file 8: Figure S8. Comparison of the intestinal microbiota in treated and post treatment mice. a Principal coordinate analysis of unweighted (left) and weighted (right) UniFrac distances of the sequences from the *TB + HRZ* (W4 to W20) and *post HRZ* (W24 to W32) animal groups described in **Figure S1**. Each sphere represents a single animal with the size of the sphere referring to the sample collection time point (early to late time points indicated as a gradient in the size of the spheres from small to large). b LEfSe analysis was performed to identify genera that are differentially abundant between the *TB + HRZ* and *post HRZ* groups. Taxa significantly enriched in the *TB + HRZ* or *post HRZ* groups depicted with orange or yellow bars, respectively. Data are filtered for *p* < 0.05 and LDA score >2. *n* = 4 for each time point. (PDF 545 kb)

Additional file 9: Figure S9. Altered coding capacity of the post treatment microbiome. PICRUSt analysis was performed on 16S sequence data to predict the KEGG pathways encoded by the microbiome of the naive and *post HRZ* (W24–W32) group animals described in **Figure S1.** LEfSe analysis was used to identify pathways that were differentially abundant between the two groups. Pathways significantly enriched in the *naive* or *post HRZ* groups depicted are with blue or yellow bars, respectively. Data are filtered for *p* < 0.05 and LDA score >2. *n* = 4 for each time point. (PDF 361 kb)

Additional file 10: Figure S10. Replicate experiment utilizing *Mtb*-infected mice for comparison of single and multi-drug effects on the microbiota. a Nine groups of mice with 3–4 animals in each group were employed. One group was left uninfected and untreated as the naïve age-matched control, and the remaining eight groups were infected with *Mtb* (aerosol). Four weeks after infection, seven of the infected groups were each treated with one or a combination of H (Isoniazid), R (Rifampin), and/or Z (Pyrazinamide) as indicated and separated by a ‘/’. b Bacterial community diversity of all the samples in each group was estimated using alpha diversity indices Chao1 (top) and Shannon (bottom). Error bars indicate maximum and minimum values. *p < 0.05; **p < 0.01; ***p < 0.001; ****p < 0.0001, Welch’s *t* test. Blue and red asterisks indicate significance in comparison to Naïve and TB groups, respectively. c Principal coordinate (PC) analysis of unweighted UniFrac distances of sequences from all nine groups. d Heat map showing the average species level relative abundance. Data shown are filtered for an overall relative variance >10 and depicted as described in Fig. 2c except along the *x*-axis, which shows the different treatment groups. Naïve, TB, HRZ, *n* = 3; remaining groups *n* = 4. (PDF 655 kb)

Additional file 11: Figure S11. Bacterial genera that are differentially abundant between the naive and each antibiotic treatment group. LEfSe analysis showing the genera significantly enriched in the comparison of the VANM versus each treatment group described in Fig. 6a. Genera significantly enriched are indicated with bars as shown in the color key. Data are filtered for *p* < 0.05 and LDA score >2. LEfSe analysis was performed without the “subclass” option. (PDF 659 kb)

Additional file 12: Figure S12. Bacterial genera that are differentially abundant between VANM and each antibiotic treatment and naive group. LEfSe analysis showing the genera significantly enriched in the comparison of the VANM versus each group described in Fig. 6a. Genera significantly enriched are indicated with bars as shown in the color key. Data are filtered for *p* < 0.05 and LDA score >2. LEfSe analysis was performed without the “subclass” option. (PDF 861 kb)

Additional file 13: Figure S13. Unweighted and weighted UniFrac analysis of the sequences from the four groups described in Additional file 1: Figure S1. Each sphere represents a single animal and all animals from all time points were included in this analysis including the samples excluded in Figs. 1b, 2b, and 5b. The size of the sphere increases with respect to time. *n* = 4–5 for each time point except W20 time point of *TB* group where *n* = 3. (PDF 1103 kb)

## Abbreviations

INH (H): Isoniazid
LDA: Linear discriminant analysis
*Mtb*: *Mycobacterium tuberculosis*
OTU: Operational taxonomic units
PZA (Z): Pyrazinamide
RIF (R): Rifampin
rRNA: Ribosomal RNA
TB: Tuberculosis
VANM: Vancomycin ampicillin neomycin metronidazole
W: Weeks
