# Supplementary figures and images for: Longitudinal profiling reveals a persistent intestinal dysbiosis triggered by conventional anti-tuberculosis therapy
Source: Microbiome. 2017 Jul 7;5:71. doi: 10.1186/s40168-017-0286-2 (PMC5501520; doi:10.1186/s40168-017-0286-2)

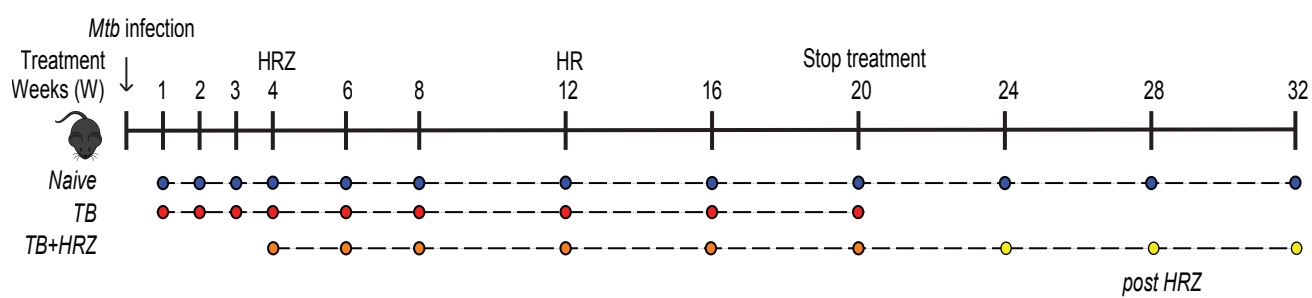

Supplement: Supplementary file 1 — Outline of experimental plan for longitudinal analysis of alterations in the microbiota induced by Mtb infection and/or ATT. Three groups of mice were employed each consisting of four to five animals (except the last time point of the TB group which consisted of three mice). For the purpose of consistency, the time points shown refer to the weeks (W) of stool sample collection relative to the date of infection rather than treatment. Fecal sample collection time points for the naïve, TB, and TB + HRZ groups are indicated with blue, red, and orange circles, respectively, along the experimental timeline. In the case of the TB + HRZ group, treatment was ceased at W20 and post treatment sampling resumed at W24 (post HRZ group, yellow circles). In addition to this experiment, two similarly designed experiments were performed to confirm the reproducibility of the key findings (see text and Additional file 6: Figure S6, Additional file 7: Figure S7). H, Isoniazid; R, Rifampin; Z, Pyrazinamide (PDF 352 kb) [file 40168_2017_286_MOESM1_ESM.pdf]

**a**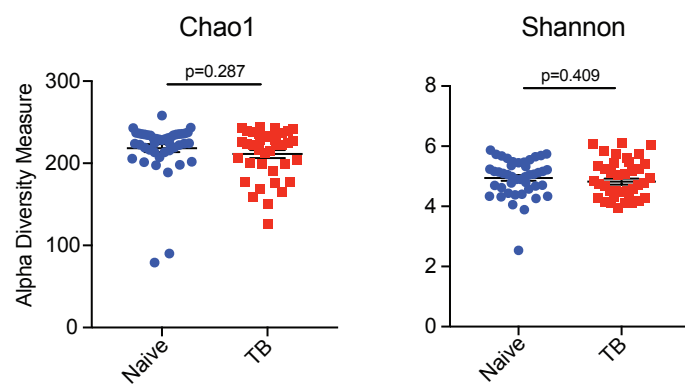**b**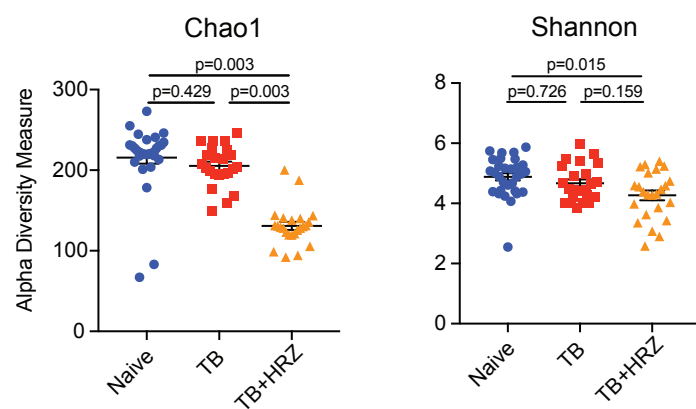**c**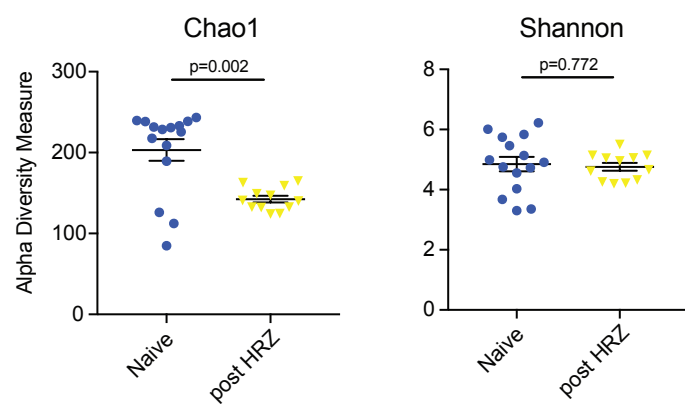

Supplement: Supplementary file 2 — Analysis of bacterial community diversity in the experimental groups shown in Figure S1. a–c Alpha diversity estimates as calculated by Chao1 (left panel) and Shannon (right panel) indices from the 16S sequence data for each of the time points in the naïve and TB (W1–W20) (a) naïve, TB, and TB + HRZ (W4–W20) (b) and naïve (W24–W32) and post HRZ (W24–W32) (c). The experimental groups are indicated along the x-axes. The bars indicate the mean ± SEM for each animal group in the comparison. Statistical significance between the groups based on pooled data from all time points of each group was calculated using a non-parametric t test with 999 Monte-Carlo permutations. (PDF 408 kb) [file 40168_2017_286_MOESM2_ESM.pdf]

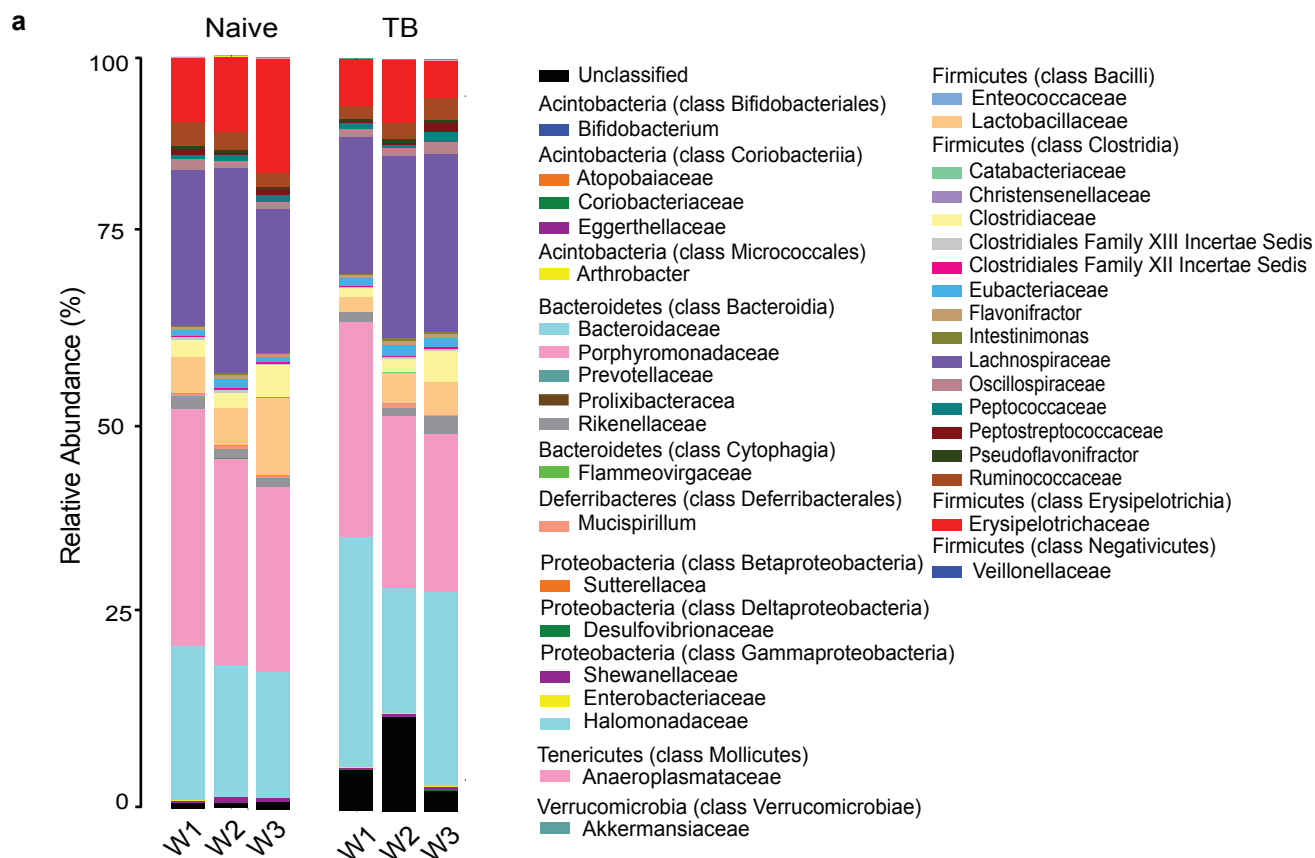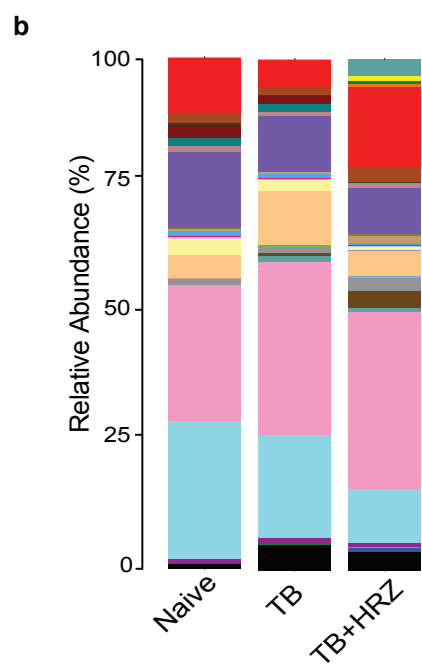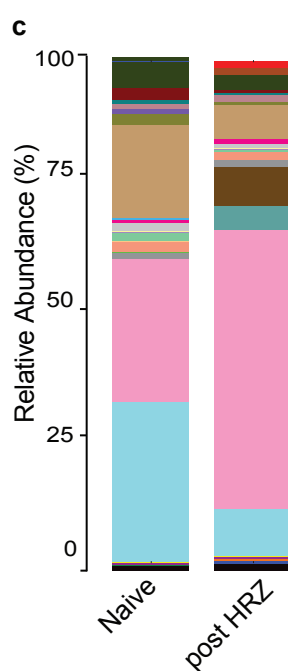

Supplement: Supplementary file 3 — Relative abundance of bacterial taxa of experimental groups. a Average relative abundance of bacterial families in the W1 to W3 time points of the naïve and TB groups. Refer Fig. 3a for the remaining time points. b, c Average relative abundance of bacterial families in naïve, TB, TB + HRZ and post HRZ groups. Averages were calculated from the sequenced data of W4–W20 time points in (b) and W24–W32 time points in (c). The bacterial families are grouped under their respective phylum and class in the color key. (PDF 451 kb) [file 40168_2017_286_MOESM3_ESM.pdf]

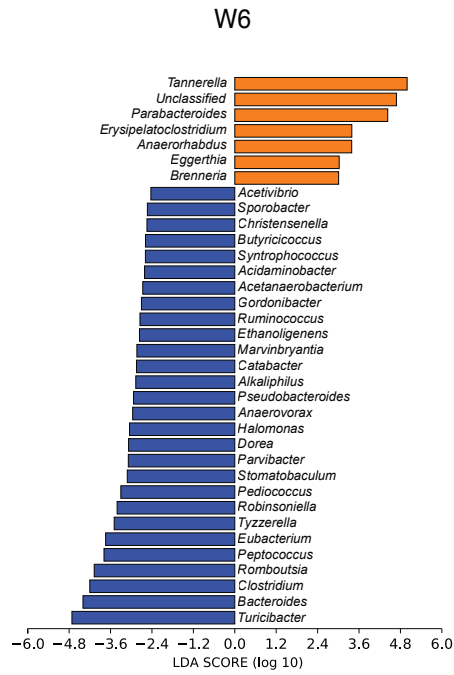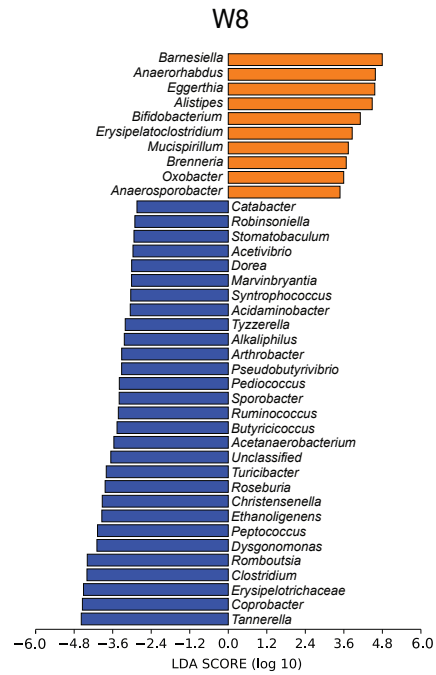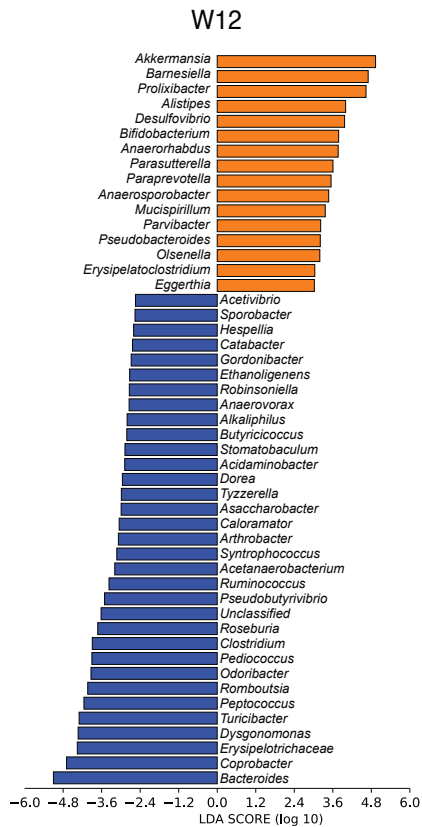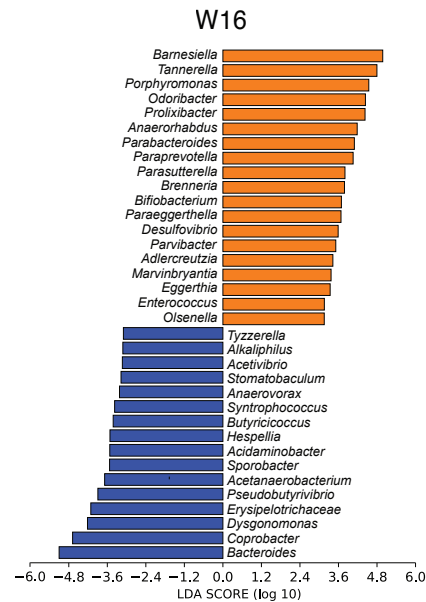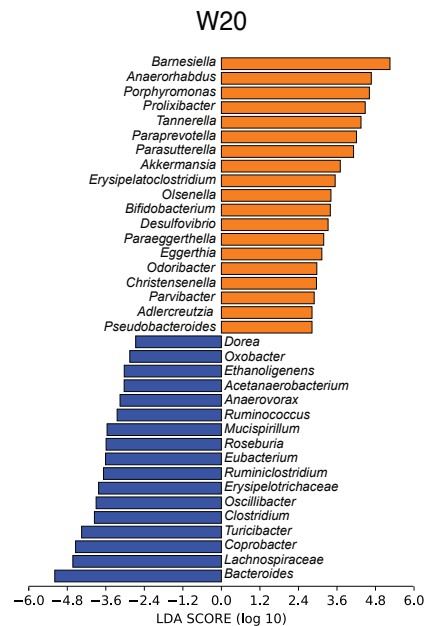

Supplement: Supplementary file 4 — Bacterial genera that are differentially abundant between the naive and TB + HRZ groups over time. LEfSe analysis showing the genera significantly enriched in the comparison of naïve versus TB + HRZ for the W6 to W20 stool collection time points as indicated. Genera significantly enriched in naïve or TB + HRZ groups are depicted with blue or orange bars, respectively. Data are filtered for p < 0.05 and LDA score >2. LEfSe analysis was performed without the “subclass” option. (PDF 656 kb) [file 40168_2017_286_MOESM4_ESM.pdf]

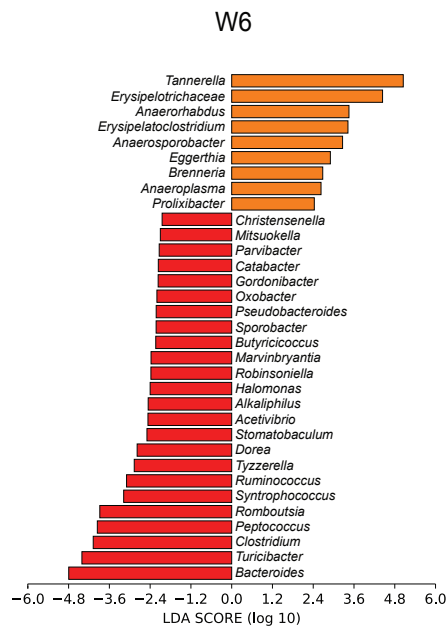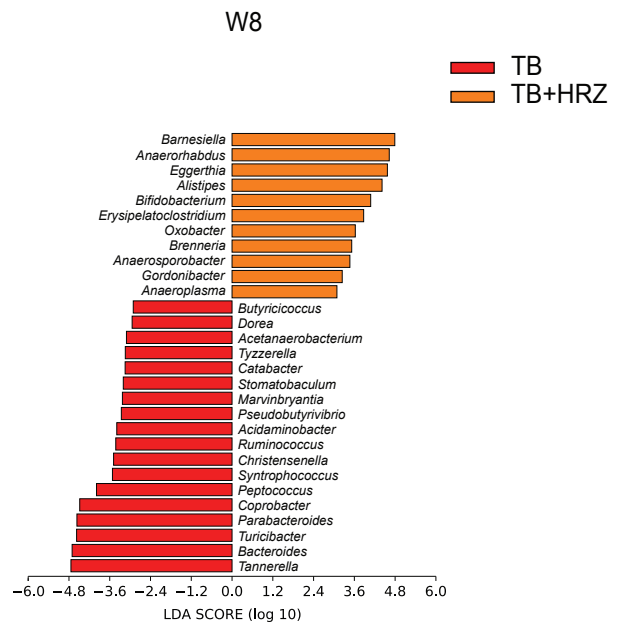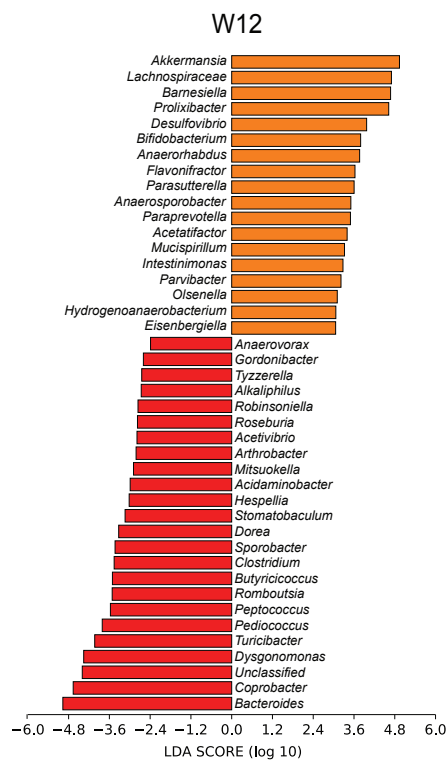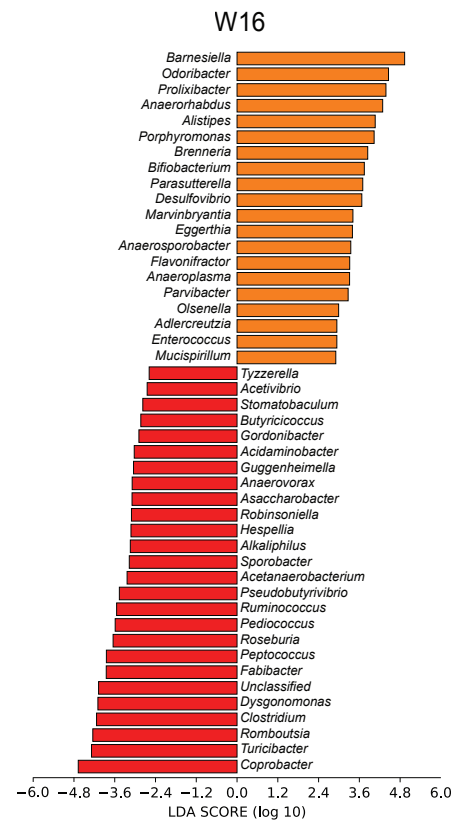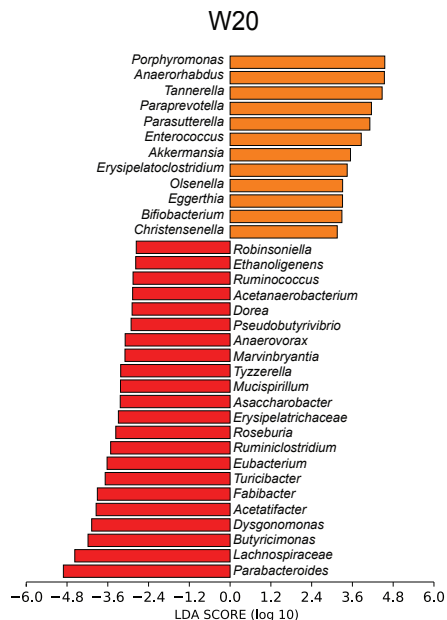

Supplement: Supplementary file 5 — Bacterial genera that are differentially abundant between the TB and TB + HRZ groups over time. LEfSe analysis showing the genera significantly enriched in the comparison of TB versus TB + HRZ for the W6 to W20 stool collection time points as indicated. Genera significantly enriched in TB or TB + HRZ groups are depicted with red or orange bars, respectively. Data are filtered for p < 0.05 and LDA score >2. LEfSe analysis was performed without the “subclass” option. (PDF 636 kb) [file 40168_2017_286_MOESM5_ESM.pdf]

a

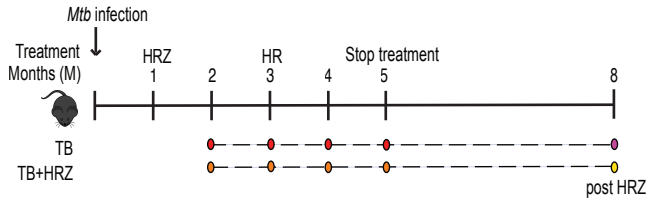

b

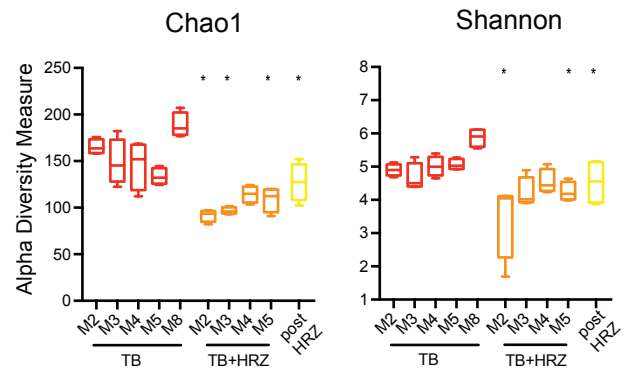

c

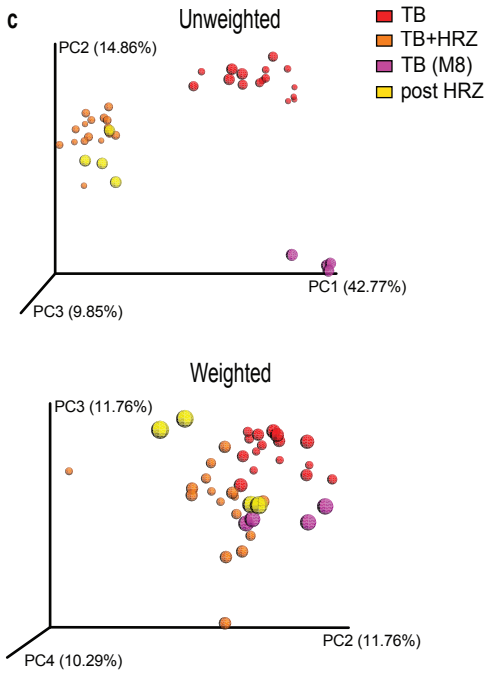

d

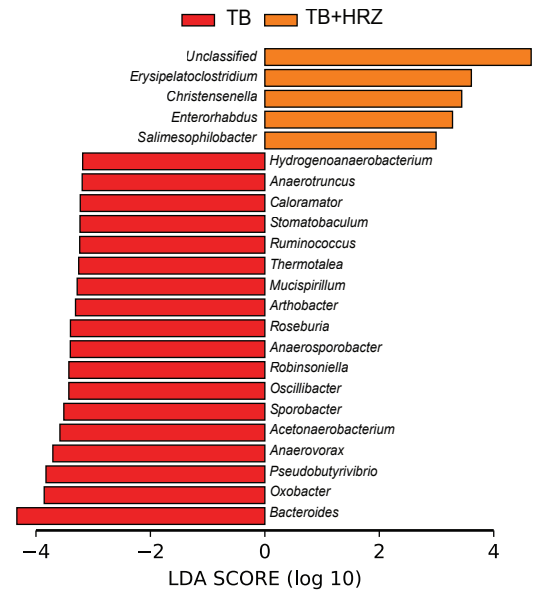

Supplement: Supplementary file 6 — Repeat experiment demonstrating reproducibility of major differences observed during as well as post treatment. a Outline of experimental plan for longitudinal analysis of alterations in the microbiota induced by ATT in Mtb-infected C57BL/6J-CD45a(Ly5a) female mice. Two groups of mice (TB and TB + HRZ) were employed with each group consisting of four animals. Stool sample collection time points are indicated as colored circles (TB, red; TB + HRZ, orange). For the purpose of consistency, the time points shown refer to the month (M) of stool sample collection relative to the date of infection rather than treatment. In the case of the TB + HRZ group, treatment was ceased at M5 and post HRZ samples (yellow circles) were collected at M8. H, Isoniazid; R, Rifampin; Z, Pyrazinamide. b Community diversity in the TB and TB + HRZ animal groups for every stool sample collected was calculated from 16S sequences using Chao1 (left) and Shannon (right) indices. Error bars indicate maximum and minimum values. Significance tests were performed between the corresponding time points in the two groups. *p < 0.05, Wilcoxon-rank sum test. c Principal coordinate (PC) analysis of unweighted (left) and weighted (right) UniFrac distances of the sequences from the animal groups. Each sphere represents a single animal with the size of the sphere referring to the sample collection time point (early to late time points indicated as a gradient in the size of the spheres from small to large). d LEfSe analysis was performed to identify genera that are differentially abundant between the TB and TB + HRZ groups. Taxa significantly enriched in the TB or TB + HRZ groups depicted with red or orange bars, respectively. Data are filtered for p < 0.01 and LDA score >2. n = 4. (PDF 719 kb) [file 40168_2017_286_MOESM6_ESM.pdf]

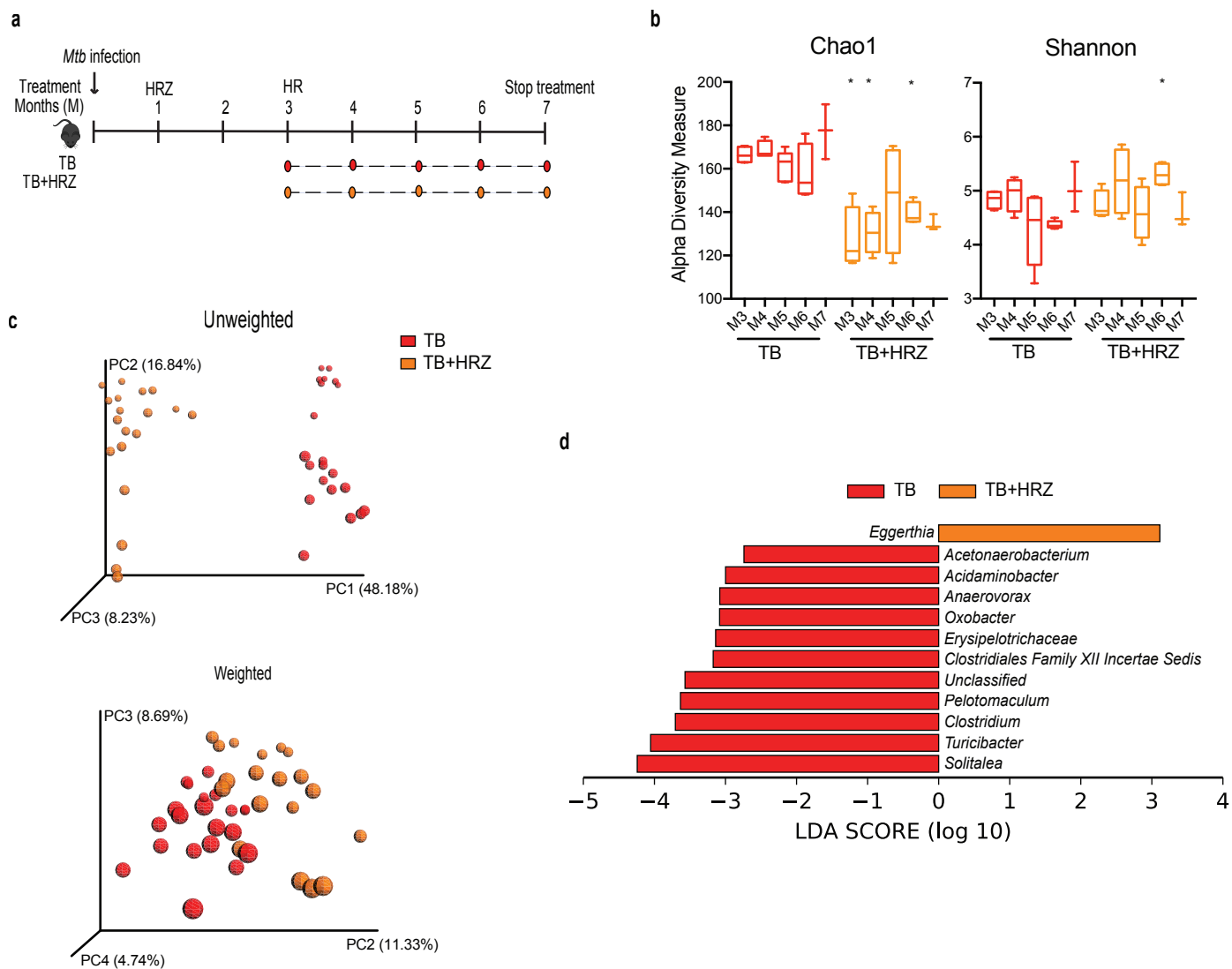

Supplement: Supplementary file 7 — Repeat experiment demonstrating reproducibility of key findings observed during treatment. See Additional file 6: Figure S6 for description with the following exceptions: C57BL/6J female mice were used and treatment was terminated at month 7 of Mtb infection, and mice were not monitored post cessation of therapy. n = 3–5. (PDF 657 kb) [file 40168_2017_286_MOESM7_ESM.pdf]

**a**

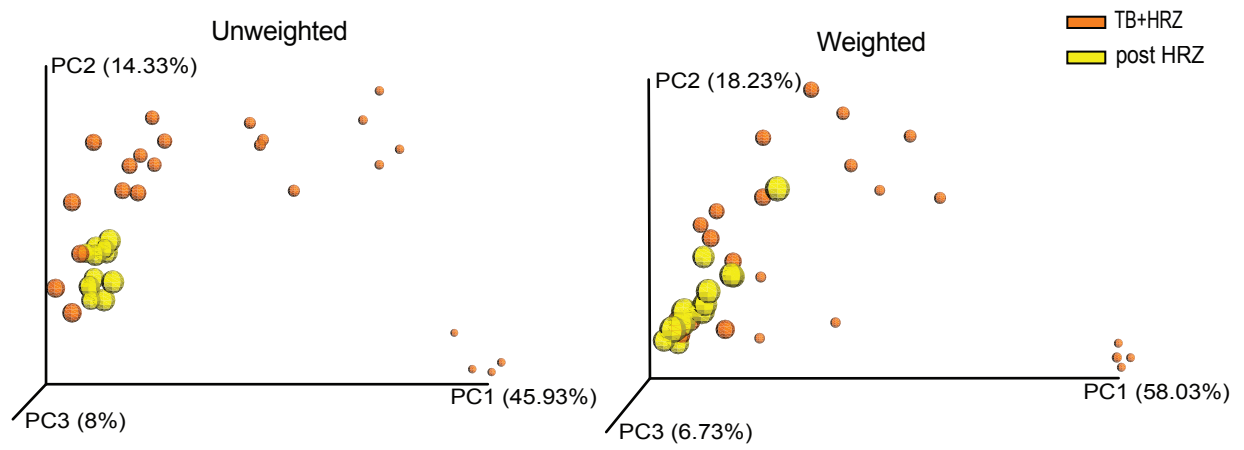

**b**

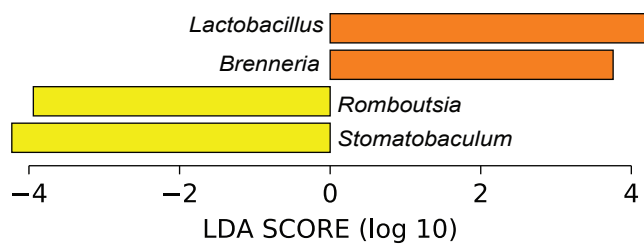

Supplement: Supplementary file 8 — Comparison of the intestinal microbiota in treated and post treatment mice. a Principal coordinate analysis of unweighted (left) and weighted (right) UniFrac distances of the sequences from the TB + HRZ (W4 to W20) and post HRZ (W24 to W32) animal groups described in Figure S1. Each sphere represents a single animal with the size of the sphere referring to the sample collection time point (early to late time points indicated as a gradient in the size of the spheres from small to large). b LEfSe analysis was performed to identify genera that are differentially abundant between the TB + HRZ and post HRZ groups. Taxa significantly enriched in the TB + HRZ or post HRZ groups depicted with orange or yellow bars, respectively. Data are filtered for p < 0.05 and LDA score >2. n = 4 for each time point. (PDF 545 kb) [file 40168_2017_286_MOESM8_ESM.pdf]

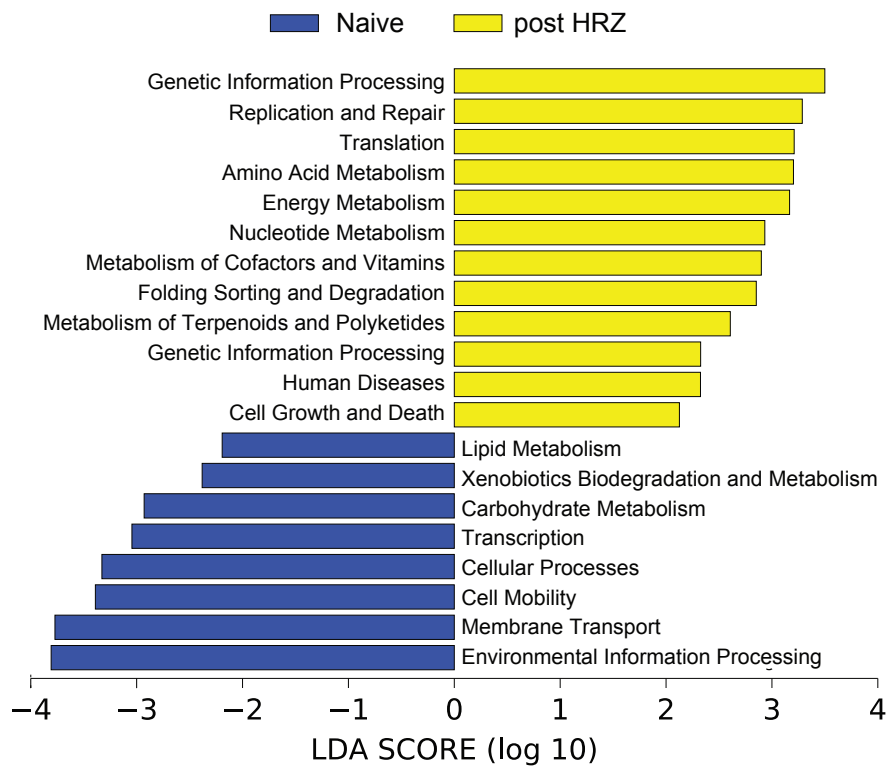

Supplement: Supplementary file 9 — Altered coding capacity of the post treatment microbiome. PICRUSt analysis was performed on 16S sequence data to predict the KEGG pathways encoded by the microbiome of the naive and post HRZ (W24–W32) group animals described in Figure S1. LEfSe analysis was used to identify pathways that were differentially abundant between the two groups. Pathways significantly enriched in the naive or post HRZ groups depicted are with blue or yellow bars, respectively. Data are filtered for p < 0.05 and LDA score >2. n = 4 for each time point. (PDF 361 kb) [file 40168_2017_286_MOESM9_ESM.pdf]

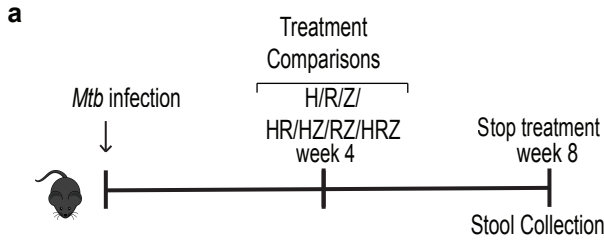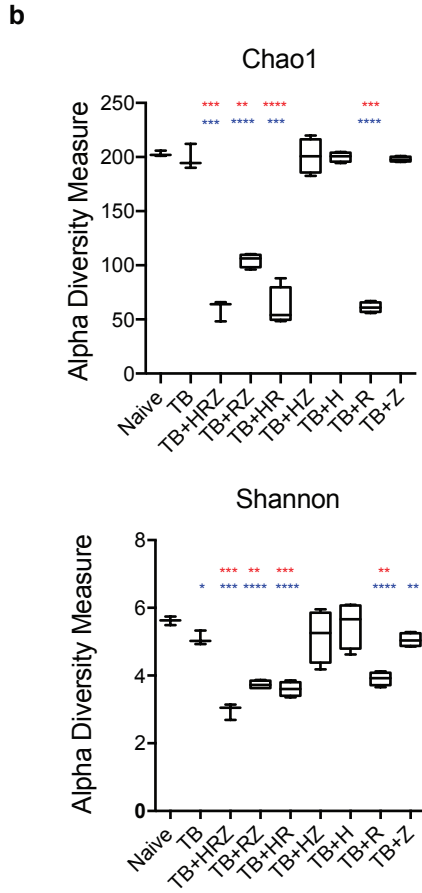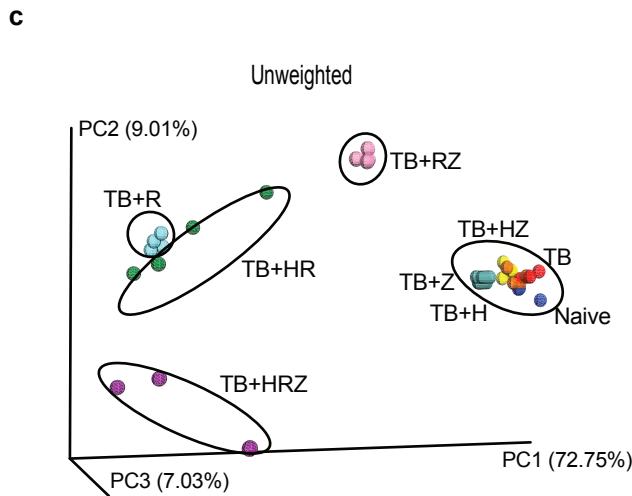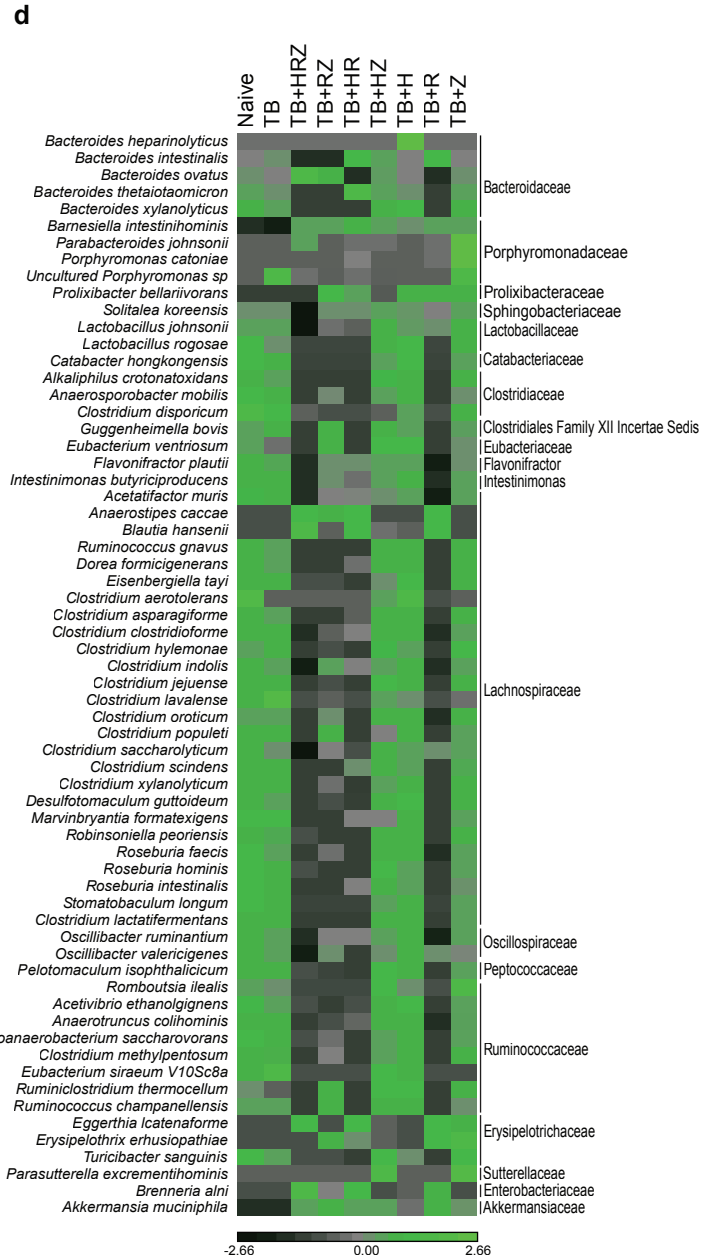

Supplement: Supplementary file 10 — Replicate experiment utilizing Mtb-infected mice for comparison of single and multi-drug effects on the microbiota. a Nine groups of mice with 3–4 animals in each group were employed. One group was left uninfected and untreated as the naïve age-matched control, and the remaining eight groups were infected with Mtb (aerosol). Four weeks after infection, seven of the infected groups were each treated with one or a combination of H (Isoniazid), R (Rifampin), and/or Z (Pyrazinamide) as indicated and separated by a ‘/’. b Bacterial community diversity of all the samples in each group was estimated using alpha diversity indices Chao1 (top) and Shannon (bottom). Error bars indicate maximum and minimum values. *p < 0.05; **p < 0.01; ***p < 0.001; ****p < 0.0001, Welch’s t test. Blue and red asterisks indicate significance in comparison to Naïve and TB groups, respectively. c Principal coordinate (PC) analysis of unweighted UniFrac distances of sequences from all nine groups. d Heat map showing the average species level relative abundance. Data shown are filtered for an overall relative variance >10 and depicted as described in Fig. 2c except along the x-axis, which shows the different treatment groups. Naïve, TB, HRZ, n = 3; remaining groups n = 4. (PDF 655 kb) [file 40168_2017_286_MOESM10_ESM.pdf]

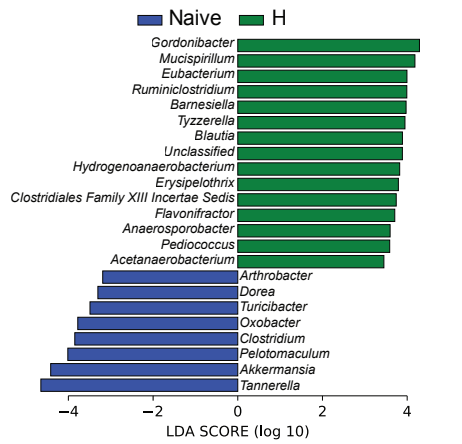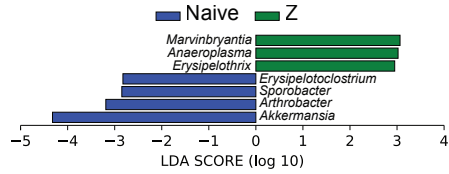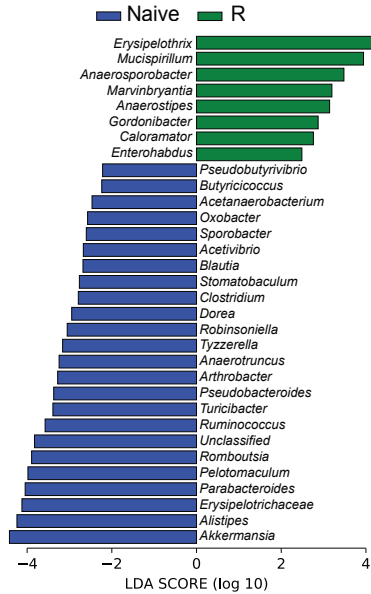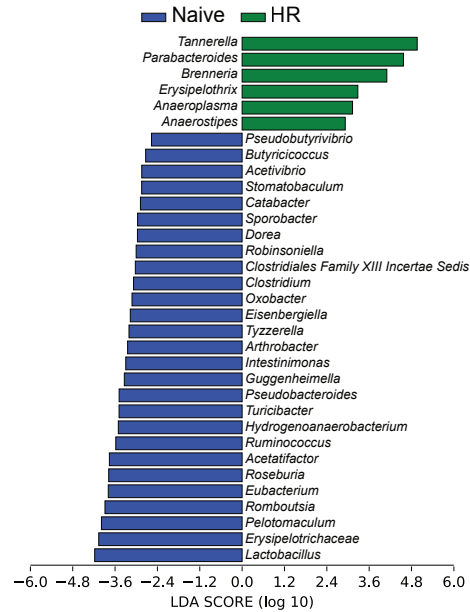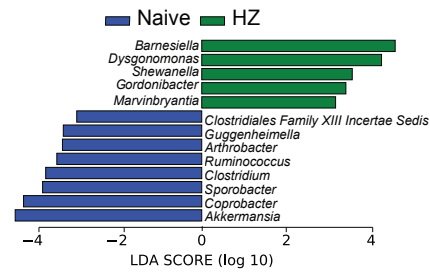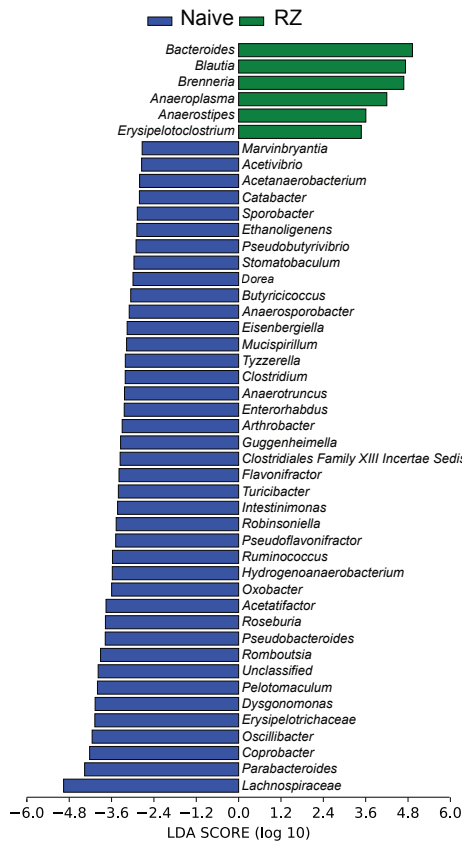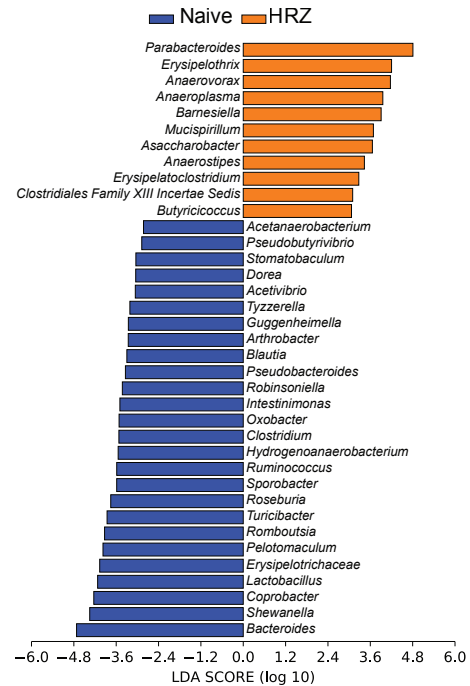

Supplement: Supplementary file 11 — Bacterial genera that are differentially abundant between the naive and each antibiotic treatment group. LEfSe analysis showing the genera significantly enriched in the comparison of the VANM versus each treatment group described in Fig. 6a. Genera significantly enriched are indicated with bars as shown in the color key. Data are filtered for p < 0.05 and LDA score >2. LEfSe analysis was performed without the “subclass” option. (PDF 659 kb) [file 40168_2017_286_MOESM11_ESM.pdf]

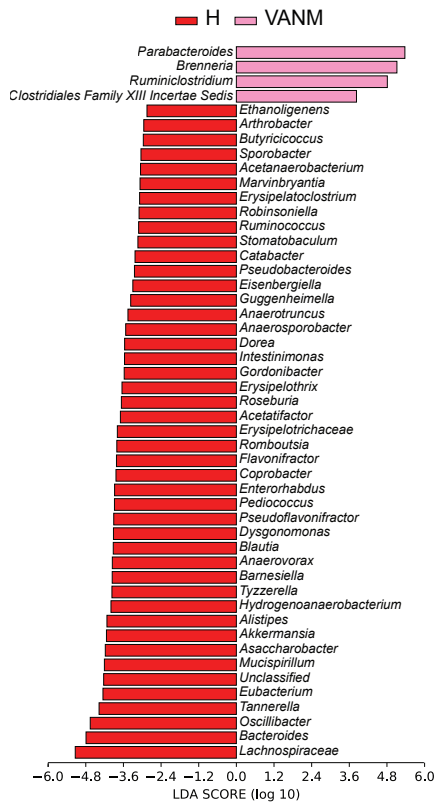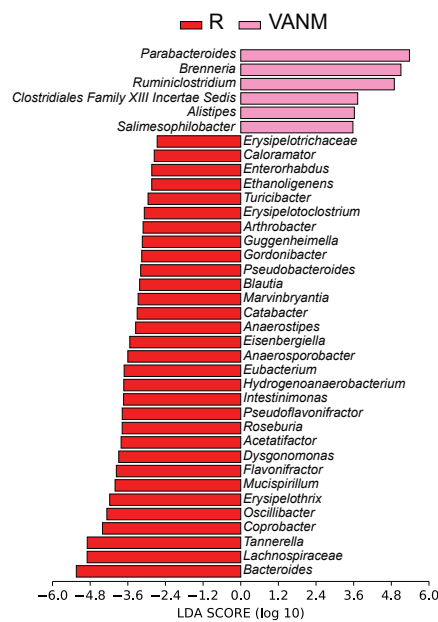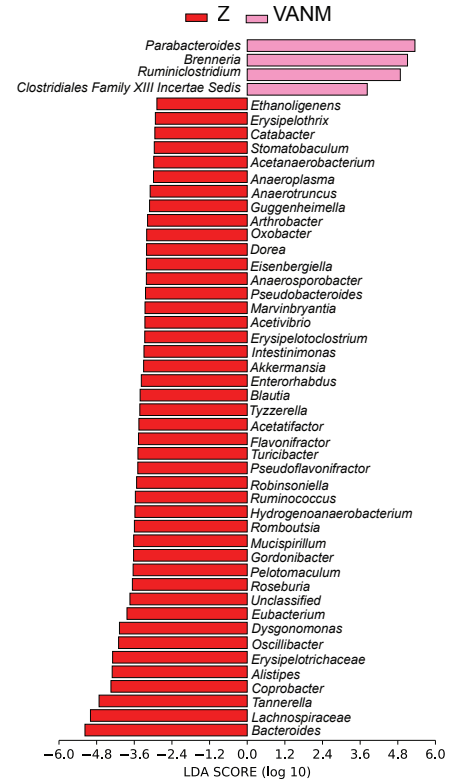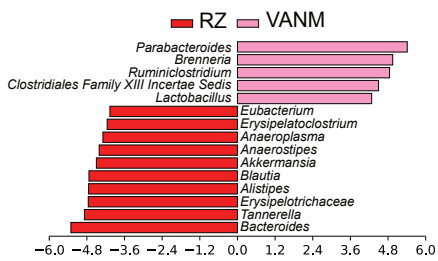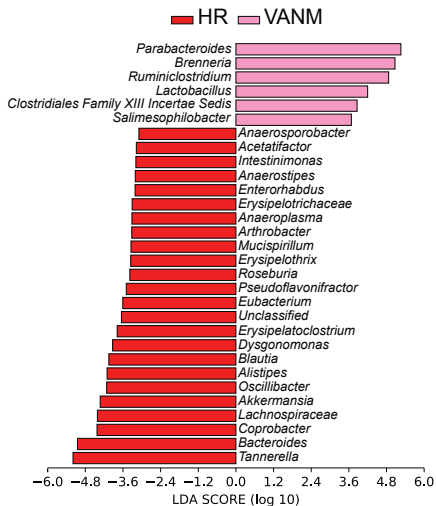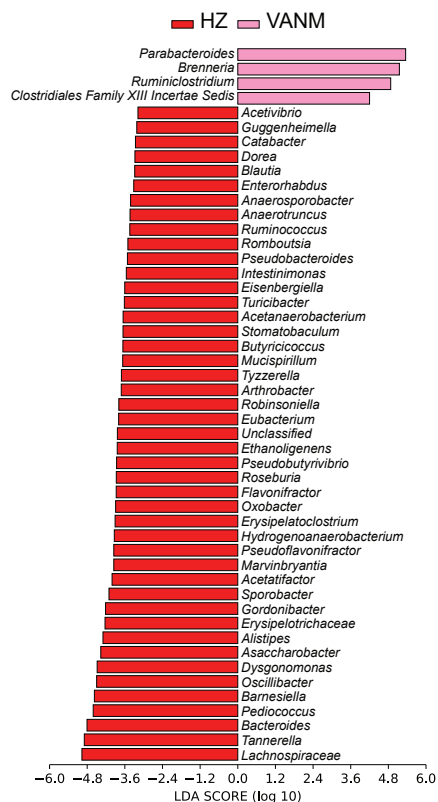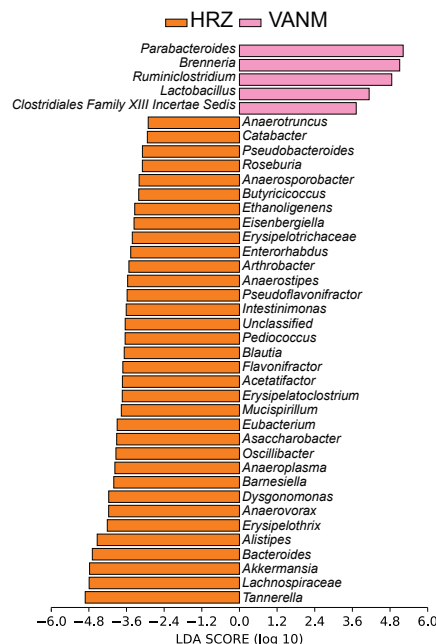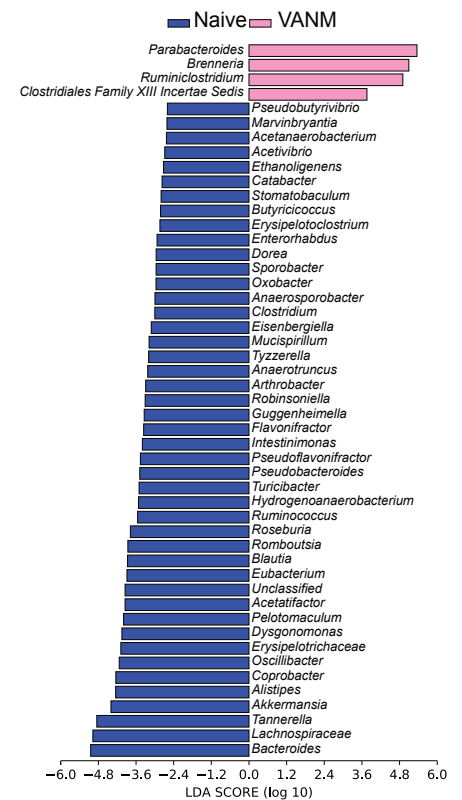

Supplement: Supplementary file 12 — Bacterial genera that are differentially abundant between VANM and each antibiotic treatment and naive group. LEfSe analysis showing the genera significantly enriched in the comparison of the VANM versus each group described in Fig. 6a. Genera significantly enriched are indicated with bars as shown in the color key. Data are filtered for p < 0.05 and LDA score >2. LEfSe analysis was performed without the “subclass” option. (PDF 861 kb) [file 40168_2017_286_MOESM12_ESM.pdf]

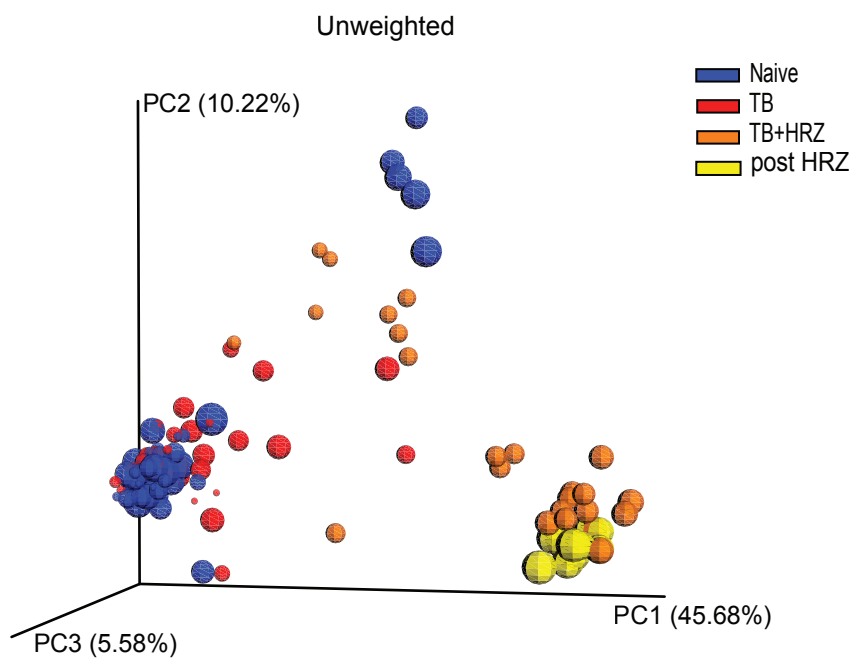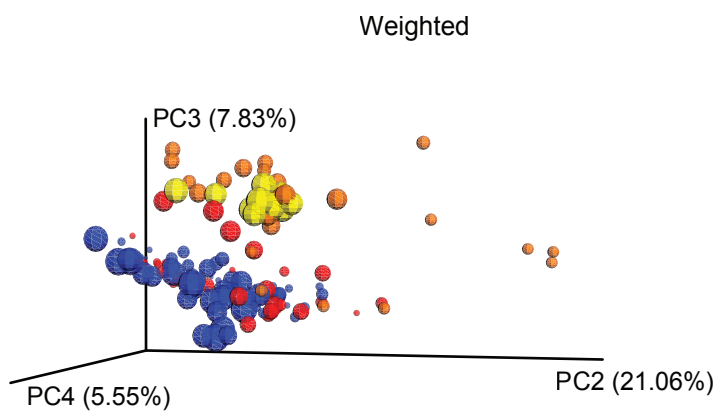

Supplement: Supplementary file 13 — Unweighted and weighted UniFrac analysis of the sequences from the four groups described in Additional file 1: Figure S1. Each sphere represents a single animal and all animals from all time points were included in this analysis including the samples excluded in Figs. 1b, 2b, and 5b. The size of the sphere increases with respect to time. n = 4–5 for each time point except W20 time point of TB group where n = 3. (PDF 1103 kb) [file 40168_2017_286_MOESM13_ESM.pdf]
